# Supplementary material for: Level of completion of maternity continuum of care among ever-married women: An analysis of Somalia’s health and demographic survey 2020
Source: PLOS Glob Public Health. 2025 Jan 10;5(1):e0004102. doi: 10.1371/journal.pgph.0004102 (PMC11723541; doi:10.1371/journal.pgph.0004102)
Supplement: Attachment — Submitted filename: Maternity CoC in Somalia_Review comments.pdf [file pgph.0004102.s002.pdf]

Review of Manuscript PGPH-D-24-00010

"Level of Completion of Maternity Continuum of Care among ever-married women: An analysis of Somalia Health and Demographic Survey 2020"

General Comments: The manuscript addresses a critical public health issue by assessing the completion levels of the maternity continuum of care among ever-married women in Somalia, utilizing data from the 2020 Somalia Health and Demographic Survey. This study is particularly relevant considering Somalia's high maternal mortality rate. The findings underscore significant gaps in maternal health service utilization, providing important insights for informing policy and programmatic interventions.

- The focus on maternal health in a high-risk region like Somalia is important, given the country's significant health challenges and elevated maternal mortality rates.
- The use of data from the 2020 Somalia Health and Demographic Survey strengthens the generalizability and relevance of the study's findings.
- The manuscript is well-structured, providing a clear and logical progression through the study's objectives, methods, results, and conclusions.

Areas for Improvement

Methods and Literature Review:

- The introduction could benefit from a more thorough review of existing literature on the maternity continuum of care, especially studies from similar contexts in Sub-Saharan Africa. This would better position the study within the broader research landscape and provide a clearer rationale.
- While the manuscript references studies from other African countries, it could enhance its analysis by offering more detailed comparisons to better contextualize the findings.
- The use of descriptive analysis is appropriate, but including more detailed statistical methods, such as logistic regression, could help identify factors associated with the completion of the maternity continuum of care, offering a deeper understanding of service utilization determinants.
- The manuscript lacks a clear explanation of how it addressed potential biases or limitations related to missing data or non-response. A more explicit discussion of these issues would strengthen the methodology section.
- The manuscript does not detail the weighting process or how weights were calculated. Providing a brief explanation of the weighting process would enhance readers' understanding of the representativeness and validity of the findings.
- A brief explanation of the rationale for restricting the analysis to "ever-married women could be helpful.

Results, Discussion, and Conclusion

- The results are clearly presented, but the manuscript could offer more detailed interpretations. For example, discussing the low completion rates of ANC4+, SBA, and PNC with insights into potential barriers and enablers would add depth.
- The manuscript acknowledges the importance of addressing rural and nomadic populations but could expand on successful strategies from similar settings or propose tailored solutions for Somalia's unique context.
- The discussion section reiterates the results but could benefit from a deeper analysis of the implications of these findings for policy and practice. For example, what specific policy changes or health system interventions are needed to improve the completion of the maternity continuum of care in Somalia?
- The discussion on corruption's impact on health services is relevant, but providing concrete examples or data would make the argument more compelling.
- The tables and figures are generally clear, but the manuscript could benefit from an additional figure or flowchart that visualizes the pathways of care, helping readers to better understand the various combinations of service utilization.
- The conclusion is concise but could be more specific in its recommendations, suggesting actionable steps for the Somali government, international donors, and NGOs working in the region.

Minor Issues:

- The citations in the main manuscript should be revised to align with PLOS Global Health referencing style.
- There are minor grammatical errors throughout the manuscript. A thorough proofreading is recommended to enhance readability.
- The references are generally well-cited, but incorporating more recent studies on the maternity continuum of care and maternal health in conflict settings would strengthen the manuscript's academic rigor.
- Some references are inaccurately attributed. For example, the manuscript incorrectly states, "This rate is much higher than the completion of ANC4 in Uganda (59.9%) ...".
- The citations in the manuscript should be revised to comply with the PLOS Global Health referencing style.

Recommendation

Minor Revisions: The manuscript is well-crafted and addresses a significant public health issue. However, it would benefit from the suggested revisions, particularly in enhancing the methodology, deepening the discussion, and expanding the literature review. I recommend that the authors implement these revisions to further strengthen the manuscript's contributions to the field.
