# Supplementary material for: Level of completion of maternity continuum of care among ever-married women: An analysis of Somalia’s health and demographic survey 2020
Source: PLOS Glob Public Health. 2025 Jan 10;5(1):e0004102. doi: 10.1371/journal.pgph.0004102 (PMC11723541; doi:10.1371/journal.pgph.0004102)
Supplement: Attachment — Submitted filename: PGPH-D-24-00010_R1_1.pdf [file pgph.0004102.s004.pdf]

PLOS Global Public Health

Level of Completion of Maternity Continuum of Care among ever-married women: An analysis of Somalia Health and Demographic Survey 2020

--Manuscript Draft--

Manuscript Number:	PGPH-D-24-00010R1
Article Type:	Research Article
Full Title:	Level of Completion of Maternity Continuum of Care among ever-married women: An analysis of Somalia Health and Demographic Survey 2020
Short Title:	Level of Completion of Maternity Continuum of Care among ever-married women
Corresponding Author:	Adam Abdulkadir Mohamed, Ph.D.<br>Baskent University: Baskent Universitesi<br>Mogadishu, SOMALIA
Order of Authors:	Adam Abdulkadir Mohamed, Ph.D.
	Ayşe Akın, Doctor
	Abdi Gele, Ph.D.
Keywords:	Continuum of care; antenatal care; Skilled birth attendant; postnatal care; Somalia
Abstract:	Introduction<br><br>Somalia has one of the worst maternal conditions in the world with maternal mortality ratio of 692 per 100,000 live births. For every maternal death, over 100 women suffer a severe obstetric morbidity. However, most of the maternal morbidity and mortality in Somalia are preventable through access to high quality care in pregnancy, and during and after childbirth. The aim of this study is to investigate the coverage and the level of completion of continuum of maternity care in Somalia.<br><br>Method<br><br>The study used a representative data from Somalia Health and Demographic Survey 2020. We restricted our analysis to ever-married women who had a live birth in the five years preceding the survey (n = 2432). The outcome variable was the completion of the continuum of maternity care (CoC). A binary variable was constructed with 'completed CoC' coded as '1' and 'not completed' coded as '0'. We analyzed the data using descriptive analyses.<br><br>Results<br><br>More than half of the women (53.1%) had their most recent births at the age ≤ 19 years old. Of all the mothers (n=2432), only 235 (9.7%) had at least four or more of the recommended antenatal care of four and above (ANC4+), 68 (2.8%) of them utilized skilled birth attendants (SBA) and 2.7% have received postnatal care (PNC) within 48hrs. In total, only 0.6% (N=14) of the women had received all of the three maternal healthcare services (ANC4+, SBA, and PNC with in 48hrs). About 78.1% of the mothers did not attend any of the three CoC services. Conclusion: Maternal health care utilization decreases as they progress from ANC4+ to PNC utilization. The government and partners should design and implement a tailored strategy to improve access and utilization of maternal healthcare services with special attention to rural and nomadic populations.
Additional Information:	
Question	Response
Financial Disclosure	The authors received no specific funding for this work.

Powered by Editorial Manager® and ProduXion Manager® from Aries Systems Corporation

Enter a financial disclosure statement that describes the sources of funding for the work included in this submission and the role the funder(s) played. This includes grants and any commercial funding of the work or authors.<br><br>This statement will be typeset if the manuscript is accepted for publication.<br><br>*Please review the submission guidelines and the instructions link below for detailed requirements and guidance.*	
**Competing Interests**<br><br>On behalf of all authors, disclose any competing interests that could be perceived to bias this work.<br><br>This statement will be typeset if the manuscript is accepted for publication.<br><br>*Please review the instructions link below and PLOS Global Public Health's competing interests policy to determine what information must be disclosed at submission.*	The authors have declared that no competing interest exist.
**Data Availability**<br><br>Before publication, Authors are required to make fully available and without restriction all data underlying their findings. Please see our PLOS Data Policy page for detailed information on this policy.<br><br>A **Data Availability Statement**, detailing where the data can be accessed, is required at first submission. Insert your Data Availability Statement in the box below.	All relevant data are within the manuscript and its supporting information files.

Please see the data reporting section of our submission guidelines for instructions on what you need to include in your Data Availability Statement.<br><br>This statement will be typeset if the manuscript is accepted for publication.<br><br>*PLOS allows rare exemptions to address legal and ethical concerns. If you have legal or ethical restrictions, please use the box below to detail these in full sentences for the Journal team to consider.*	

Powered by Editorial Manager® and ProduXion Manager® from Aries Systems Corporation

Manuscript
Click here to access/download;Manuscript;CoC Manuscript_revised_27-09-2024.docx

Level of Completion of Maternity Continuum of Care among ever-married women: An analysis of Somalia Health and Demographic Survey 2020

Adam A. Mohamed[1,2*], Ayşe Akın[2], Sare Mihciokur[2], Sarp Üner[3], Abdi Gele[4]
[1] Department of Program Quality & Development, Save the Children, Mogadishu, Somalia
[2] Department of Public Health, Faculty of Health Sciences, Başkent University, Ankara, Turkey
[3] Department of Public Health, Faculty of Medicine, Lokman Hekim University, Ankara, Turkey
[4] Department of Research, Norwegian Institute of Public Health, Oslo, Norway
***Adam Abdulkadir Mohamed** Correspondence: Adamafrican@gmail.com

Abstract

Introduction: Somalia is continuing its retrieval from three decades of underdevelopment, political instability, civil unrest, and protracted humanitarian crises. Somalia has one of the worst maternal conditions in the world. For instance, the maternal mortality ratio is 692 per 100,000 live births. Extra efforts are needed to improve maternal health. In this study, we aim to investigate the level of completion and coverage along maternity continuum of care in Somalia

Method: The study used data from Somalia Health and Demographic Survey 2020. We restricted our analysis to ever-married women who had a live birth in the five years preceding the survey (n = 2432). Completion of the continuum of maternity care was the outcome variable for this study. It was constructed into a binary variable with complete coded as 1 and incomplete coded as 0. We categorized it into three models: ANC4+ as the first model, ANC4+ & SBA as the second model, and ANC4+ & SBA & PNC as the third model.

Results: More than half of the women (53.1%) had their most recent births at the age ≤ 19 years old. of all the mothers (n=2432), only 235 (9.7%) had at least four or more of the recommended antenatal care (ANC4+), 68 (2.8%) of them utilized skilled birth attendants and only 14 (0.6%) of women had received all of the three maternal healthcare services (ANC4+, SBA, and PNC with in 48hrs). About 78.1% of the mothers did not attend any of the three CoC services.

Conclusion: Maternal health care utilization decreases as they progress from ANC4+ to PNC utilization. The government and partners should design and implement strategies to improve maternal healthcare utilization specific to [illegible]al and nomads, less educated, not working, low income, and less power to decision-making.

Keywords: Continuum of care, Antenatal Care, Skilled birth attendant, Postnatal care, Somalia

1. Background

Sub-Saharan Africa (SSA) has the highest rates of maternal mortality in the world, accounting almost 90% of the global burden of maternal mortalities (1, 2). This high burden of maternal mortality in the region has been attributed to preventable factors such as low antenatal care (ANC) utilization, low uptake of skilled attendant delivery and postnatal care (PNC) (3). In 2020, the MMR in the African Region was 531 deaths per 100 000 live births, accounted for 69% of global maternal deaths in 2020 (4). From 2000 to 2020 the maternal mortality ratio dropped by about 34% worldwide. However, in many low- and middle-income countries maternal mortality remains a significant public health problem with nearly 94% of all maternal deaths occurring in low resource settings to date (5).

Somalia is continuing its retrieval from three decades of underdevelopment, political instability, civil unrest, and protracted humanitarian crises. The country has suffered multiple emergencies including flooding, drought, famine, locust attacks, other climate change shocks which resulted in many deaths and large-scale population displacements (6). Somalia has a population of 17 million, with 44 percent of the population living in urban areas, 23 percent living in rural areas, 26 percent in nomadic areas, and 9 percent living in Internally Displaced settings (IDPs) (7). The country has one of the weakest health care systems in Sub-Saharan Africa, due to limited state capacity, poverty-related deprivation, corruption, and longstanding armed conflicts. Therefore, Somalia has some of the lowest health indicators in the world (6). The fragile health system in Somalia is shaped by various administrations that adopted different policies, priorities, and health care service approaches, often influenced by local state administrations and international paradigms and resolutions (8).

Somalia is among the 15 countries that WHO marked as very high alert countries for maternal, newborn, and under 5 deaths. Most of the causes are either preventable or treatable. Somalia has one of the worst maternal [illegible]ditions in the world. For instance, the maternal mortality ratio is 692 per 100,000 live births. Poor maternal health care delivery in the rural communities results the majority of the maternal, newborn, and child deaths during pregnancy, childbirth, and after delivery in Somalia (9). This is due to the unavailability and low utilization of maternal healthcare services including skilled birth attendance, emergency obstetric care, postnatal care, and family planning. According to a predictive analysis on the trends of maternal mortality ratio, Somalia will not meet the sustainable development goal target 3.1 of reducing the maternal mortality ratio to

less than 70 per 100,000 live births. The country will not also meet the targets in reducing neonatal mortality to below 12 per 1000 live births and under-5 mortality to below 25 per 1000 live births by 2030 (10). According to Somalia's Voluntary National Reviews Report 2022 and voluntary national reviews in East African countries on Sustainable Development Goals specially goal 3, Somalia still faces significant challenges in [illegible]ernal mortality rates, which can be attributed to low uptake of antenatal care and postnatal care and a low number of deliveries at health facilities or with skilled health care providers. Somalia is unlikely to achieve the SDGs given the lack of evidence-based knowledge that inform government interventions to improve maternal and newborn health (11).

Continuum of Care (CoC) refers to the continuity of care throughout pregnancy, birth, and after delivery [illegible]., use of antenatal care, skilled birth attendance, and postnatal care). Ensuring that continuity of care for maternal, newborn, and child health has become a key for improving the health of the mothers, newborns, and children. The continuum of care has newly been emphasized as a core principle of programmes for maternal, newborn, and child health, and as a method to reduce the burden of the maternal, newborn, and child deaths (12).

The main services for continuum of care like antenatal care, facility delivery, and postnatal care are recognized to reduce maternal and child mortality and morbidity in high burden settings. Maternal and child mortality rates are indicators that are used globally to determine the health, economy, and developmental status of countries. Although the Somalia government has taken a significant step to increase the availability and access of the maternal care, still a low proportion of mothers utilize the services specially the urban areas (13). Improving the utilization of the maternity continuum of care relies on a better understanding of the barriers and gaps affecting the uptake to each service i.e., ANC4+, SBA, and PNC. In this study, we aim to investigate the level of completion and coverage along maternity continuum of care in Somalia.

2. Methods

2.1. Data source and study participants

This study used data from the 2020 Somalia Health and Demographic Survey which is a nationally representative household survey that was designed to collect, analyze, and disseminate demographic data on reproductive health, maternal and child mortality, family planning and fertilities, nutrition, and health care utilization in Somalia. The main objective of the Somali Health and Demographic Survey was to provide evidence on the health and demographic characteristics

of the Somali population that will guide the development of programmes and formulation of effective policies. This nationally representative survey used a multistage cluster sampling design to collect data on reproductive health, maternal and child mortality, family planning and fertilities, nutrition, and health utilization in Somalia. About 11,884 ever-married women of reproductive age (15-49) completed the interviews. Our study focused on ever-married women who had a live birth in the five years preceding the survey (n=2432) as you can see in figure 1 below. The survey further restricted the women's most recent live births in the recall period. 9,470 were not eligible due to either not having children less than 5 years or incomplete data in the maternal and child health questions or not fitting the targets and the analysis removed them. Marriage is associated with childbearing and women can have a child only if she is married as accepted in the community. That is why we restricted our study on only the ever-married women.

2.2.Data analysis

We used STATA software (version 18) and sampling weight for all analysis with survey data analysis command (svy) to account for the cluster survey design and missing responses. The study did not proportionally sample on urban, rural, and nomadic domains hence urban overrepresentation may occur. Large part of the population refused to finalize the questionnaires specially the maternal and child health section, hence causing incomplete interviews. The SHDS data have a missing information due to some aspects of the population were not covered. The SHDS data in Somalia was not meant to tract detailed aspects of maternity care which is making difficult to analyze the maternity continuum of care. We checked data for completeness and then cleared. We then conducted a descriptive analysis (number, frequency, and percentage) to summarize the characteristics of the study population and each variable. We assessed the level of coverage for each maternal health service (ANC, SBA, and PNC) separately and then together as a combined continuum of maternity care. Variance Inflation Factor was used to test the presence of collinearity between the independent variables using STATA 18.

3. Results

3.1.Characteristics of the study participants

Table 1 summarizes the background characteristics of ever-married mothers included in this study. The mean age of the women in the study was 22.8 years (SD = 4.2) with more than half of the women (53.1%) had their most recent births at the age of ≤19 years while 90.2% of the women in the study were married during the survey period. Above sixty percent of the women were living in

urban area and seventy-seven had no education. 96.1% of the women were not working, 74.5% of them had 1-2 children, and 90.6% had not accessed to mass media (radio exposure). Wealth quintile distribution among the households were almost homogenous proportion with the highest being the poorest (25.5%) and the lowest being the middle group (16.7%) as you can see the below table 1.

Table 1: Socio-demographic characteristics of ever-married women who had at least one a live birth in the five years preceding the survey (Weighted sample size = 2432 & unweighted sample size = 2414)

Variable	Categories	Weighted Number (2432)	Percentage (%)
**Maternal age at birth (years)**	<20	1,291	53.1
	20-34	1,125	46.3
	35-49	16	0.6
**Birth Order**	1	906	37.3
	2	908	37.3
	3	525	21.6
	4 or more	93	3.8
**Residence**	Urban	1,475	60.6
	Rural	653	26.9
	Nomadic	304	12.5
**Marital Status**	Married	2,193	90.2
	Divorced/widowed	239	9.8
**Mother's Education**	No Education	1,872	77.0
	Primary	370	15.2
	Secondary & above	190	7.8
**Mother's Employment**	Working	96	3.9
	Not working	2,336	96.1
**Number of Children**	1-2	1,813	74.5
	3-4	598	24.6
	>=5	21	0.9
**Decision-making power in determining own healthcare**	Women or jointly	1,285	52.8
	Husband or others	1,147	47.2
**Exposure to Radio**	Yes	229	9.4
	No	2,203	90.6
**Wealth Quintile**	Poorest	621	25.5
	Poorer	486	20.0
	Middle	406	16.7
	Richer	454	18.7
	Richest	465	19.1

3.2.Overall use of maternal health services in Somalia

Table 2 shows the descriptive analysis indicating that Somalia did not achieve a good antenatal care, institutional delivery, and postnatal care coverage. Around **two-third** of the study participants (66.2%) did not receive antenatal care visits, and only 24.2% received ANC between 1-3 visits

whereas 9.7% of the women in the study attended the recommended four or more antenatal care (ANC4+) visits. Less than 14% of the deliveries was attended by skilled provider. For postnatal care, only 2.7% of the women had a PNC check within 48 hours after delivery regardless of their delivery place.

Characteristics	Categories	Weighted Number (2432)	Percentage (%)
ANC visits	No ANC	1,608	66.2
	1-3ANC	589	24.2
	ANC4+	235	9.7
Delivered by SBA	Skilled provider	327	13.4
	Unskilled provider	1,478	60.8
	No one	47	1.9
	Don't know/missing	580	23.8
PNC visits	First 48hrs	67	2.7
	More than 48hrs	2	0.1
	No PNC/Don't know	2,363	97.2

Table 2: Number and Percentage of mothers and their use of maternal health services.

Maternity Continuum of Care: of all the mothers (n=2432), only 235 (9.7%) had at least four or more of the recommended antenatal care (ANC4+) visits during their recent pregnancy and among this, only 9 mothers live in nomadic settings. Out of the women who have received four or more ANC care, only 68 (2.8%) of them utilized skilled birth attendants in their most recent pregnancies. Regarding continuum of maternal health services, only 14 (0.6%) of women had received all of the three maternal healthcare services (ANC4+, SBA, and PNC with in 48hrs).

1.1.Pathways of maternal healthcare use

Considering the study's three main outcomes (ANC4+, SBA, and PNC), we created eight different pathways or combinations of maternal health service utilization i.e., from not receiving any of the three maternal health services to complete use of all three maternal health services (Table 3). More than two-third of the mothers in the study (78.1%) did not receive any of the three maternal health services as shown in pathway 1. Notably, only 14 mothers (0.6%) received all of the three maternal health services along the continuum (pathway 8). Three groups that attended any two of the three maternal health services (pathway 3, 6, and 7) accounted for 2.2%, 0.5%, and 0.1% respectively. Lastly, the three groups that utilized only one of the three maternal services (pathways 2, 4, and 5) accounted 6.7%, 1.6%, and 10.2% respectively.

Pathway	ANC4+	SBA	PNC within 48hrs	Frequency (Weighted Number)	Percentage
1	–	–	–	1,899	78.1

2	+	–	–	164	6.7
3	+	+	–	54	2.2
4	–	–	+	39	1.6
5	–	+	–	248	10.2
6	–	+	+	11	0.5
7	+	–	+	3	0.1
8	+	+	+	14	0.6
**Total**				**2,432**	**100%**

Table 3: Percentage distribution of the three maternal health services received by women. **+** *Received the service;* **-** *Did not receive the service.*

The table 3 above shows the combination of maternal health services (three major services) that mothers either received or did not receive. It is summarizing the three major outcome variables of the study i.e., antenatal care of four times or above, skilled birth attendant, post-natal care within 48 hours after delivery.

2. Discussion

This study assessed maternity continuum of care for the first time in Somalia. Access and use of maternity care services during pregnancy, childbirth, and the postnatal period from skilled providers are essential for the survival and wellbeing of the mother and newborn. It is particularly critical in setting where teenage pregnancy is common such as Somalia. Our study states that 53% of women gave birth their first child at the age of ≤ 19, while the completion of ANC4 was only 9.7%. This rate is much higher than the completion of ANC4 which is 59.9% in Uganda (15), and that of Ethiopia which is 33% (16). Protracted conflicts and instability may explain why women in Somalia have low prevalence of ANC4. Armed conflict has been described as an important contributor to persistent excess maternal and child deaths while it can severely reduce access to maternal health services and thus lead to poor maternal health outcomes (17). The effect of the three decades of conflict on the access to health care in Somalia is exacerbated by the pervasive corruption in the health sector, given the fact that systemic corruption and the large-scale misappropriation of state funds is the norm in Somalia (18). Somalia, like many other countries, corruption at the health sector range from smaller-scale acts by doctors and nurses who charge "informal payments" to larger-scale acts at the ministerial level, when people in power employ incompetent relatives or redirect resources away from those who need it for their own benefit (19). Hence, countries with high levels of corruption spend less on health care (20). In addition, high levels of corruption correlate with higher infant and child mortality rates (21). To improve maternal

health care in Somalia, requires political and economic commitment, and genuine leadership is critical in achieving this agenda.

Our study shows that only 13.4% of women in Somalia gave birth with the help of skilled health provider. A prior study on 29 Sub-Saharan African countries found an average proportion of women who had skilled assistance during delivery was 75.3%, ranging from 38.4% in Chad to 93.7% in Rwanda (3). Skilled attendants have positive contribution in the reduction of maternal and newborn mortality and morbidity. A 40% decline in maternal deaths in SSA occurred between 2000 and 2017 was attributed, by WHO, to utilizing skilled birth attendant at delivery (22). The fact that only 13.4% of women in Somalia are using the services of skilled birth attendant, indicated that although utilization of the services of SBAs in SSA appears high, it is [illegible]remely low in Somalia. [illegible] SBA [illegible]valence in Somalia can be improved by adopting prevailing successful interventions of countries with SBA successes such as Rwanda, whilst considering of contextual variations (23). Our study finds that the overall completion of maternity continuum of care is less than 1%. This is much lower than that reported in Gondor Ethiopia 47% (24). This could be due to the efforts made by the Ethiopian government to in the recent years to strengthen maternity health care access.

Our study found that only 2.8% of Somali women have received postnatal care. Receiving postnatal care after delivery is vital for women and the baby due to the fact that over 65% of maternal and neonatal deaths occur during the first 42 days of postpartum and during the first 7 days of life [illegible]ectively. A study on postnatal care utilization in 36 sub-Saharan Africa countries found a prevalence of PNC of 52.48%[illegible]% CI: 52.33, 52.63], ranging from 73.51% in Central Africa region to 31.71% in Eastern Africa Region (25). A prior study found an association of armed conflicts with an increase of 36.9 maternal deaths per 100,000 live births, an increase of 2.8 infant (under 1 year old) deaths per 1,000 live births (17).

The continuity of maternity health care services is the care that a woman uses the three recommended cares of antenatal care (ANC), skill birth attendant (SBA), and postnatal care (PNC). Our study shows that only 0.6% of the women in Somalia have completed the three services; ANC4, SBA and PNC, while only 2.8% have completed ANC4 and SBA. The prevalence

of maternal continuum of care in Somalia which is stated by our study (0.6%), is far much lower than the frequence of continuum of care in Ethiopia (47%) (24) and is even over 15 times lower than the continuum of care of rural women in Ethiopia (13%) (26). Further, our study shows a significant inequity in continuum of care by residence with zero continuum of care among women in pastoral communities that constitute 26% of total population in Somalia (7). A recent study in Somalia stated that pastoralist women have limited access to maternal health care which supports our findings (13). The inequity between urban and rural women, in utilizing maternal health care services, is prevalent across Africa. A prior study of 27 African countries showed a prevalence of ANC utilization of 34.7% among urban women and 22.4% among in rural areas, while SBA prevalence was nearly 90% and 69% in urban women and rural women respectively (27).

This study has both strength and limitations. The main strength of this study is the use of weighted nationally representative data with a large sample, which makes it representative at national level. The SHDS data used a multi-stage cluster sampling design, and some groups or demographic categories might be over sampled hence, we weighed the SHDS data during analysis.

The current SDHS did not collect information on type of provider for each visit, therefore, we couldn't determine the type of provider for each visit. Further, approximately 2.6 million Somalis are currently displaced within their own country and the SHDS 2020 did not include the IDP domain in the data.

Conclusion.

The prevalence of completion of the maternity continuum of care was found to be lower than any country in Africa. Maternal health care utilization decreases as they progress from ANC4+ to PNC utilization. Inequity in the continuum of care is a chronic problem in Somalia where pastoralist women who constitute 26% of the population have zero completion of continuum of care. Health inequalities are avoidable because they are rooted in political and social decisions. Political and economic commitment is required in improving the access to maternal health care in the country. The findings of our study indicate that if the government does not target the rural and pastoralist communities, women's retention to the health services if existing would be minimum.

To reduce health inequalities between urban and pastoralists in Somalia, we need to act across a range of health-policy areas including policies to improve access to maternal health care in pastoralists setting in the country. The government and partners should design and implement strategies to improve maternal healthcare utilization specific to rural and nomads, less educated,

not working, low income, and less power to decision-making. [illegible] government of Somalia should invest and increase the number of certified skilled birth attendants i.e., nurses, midwives, and obstetricians to provide safe and competent care during childbirth. To attract and retain skilled birth attendants in remote and rural areas, the government and its partners should implement incentive programs designed to the professional and certified midwives and obstetricians and should train and deploy community health extension workers in rural areas to provide basic maternal health educations, identify pregnant women, and support and encourage them to seek health care.

Data Availability Statement: All relevant data are within the manuscript and its supporting information files.

Funding: The authors received no specific funding for this work.

Competing interest: The authors have declared that no competing interest exist.

References

1. Yaya S, Anjorin SS, Adedini SA. Disparities in pregnancy-related deaths: spatial and Bayesian network analyses of maternal mortality ratio in 54 African countries. BMJ global health. 2021;6(2):e004233.
2. Onambele L, Ortega-Leon W, Guillen-Aguinaga S, Forjaz MJ, Yoseph A, Guillen-Aguinaga L, et al. Maternal mortality in Africa: Regional trends (2000–2017). International journal of environmental research and public health. 2022;19(20):13146.
3. Budu E, Chattu VK, Ahinkorah BO, Seidu A-A, Mohammed A, Tetteh JK, et al. Early age at first childbirth and skilled birth attendance during delivery among young women in sub-Saharan Africa. BMC pregnancy and childbirth. 2021;21:1-12.
4. Ward ZJ, Atun R, King G, Dmello BS, Goldie SJ. Global maternal mortality projections by urban/rural location and education level: a simulation-based analysis. Eclinicalmedicine. 2024;72.
5. Organization WH. Trends in maternal mortality 2000 to 2020: estimates by WHO, UNICEF, UNFPA, World Bank Group and UNDESA/Population Division: World Health Organization; 2023.
6. Mackey KP, Kiptum SE. Health systems strengthening in fragile contexts: A partnership model in South West State, Somalia. Field Exchange 57. 2018:89.
7. Federal Republic of Somalia. POPULATION ESTIMATION SURVEY 2014 SOMALIA FOR THE 18 PRE-WAR REGIONS OF SOMALIA. 2014. chrome-extension://efaidnbmnnnibpcajpcglclefindmkaj/https://somalia.unfpa.org/sites/default/files/pub-pdf/Population-Estimation-Survey-of-Somalia-PESS-2013-2014.pdf.
8. Warsame AA. Somalia's Healthcare System: A baseline study & human capital development strategy. Heritage Institute and City University of Mogadishu. 2020.
9. Aden JA, Ahmed HJ, Östergren P-O. Causes and contributing factors of maternal mortality in Bosaso District of Somalia. A retrospective study of 30 cases using a Verbal Autopsy approach. Global health action. 2019;12(1):1672314.

10. Morrison J, Malik SMMR. Population health trends and disease profile in Somalia 1990–2019, and projection to 2030: will the country achieve sustainable development goals 2 and 3? BMC Public Health. 2023;23(1):1-9.
11. Chisika SN, Yeom C. Voluntary National Reviews on Implementation of Sustainable Development Goals: Key Lessons from East African Member States. SAGE Open. 2024;14(2):21582440241257319.
12. Kerber KJ, de Graft-Johnson JE, Bhutta ZA, Okong P, Starrs A, Lawn JE. Continuum of care for maternal, newborn, and child health: from slogan to service delivery. Lancet. 2007;370(9595):1358-69.
13. Duale HA, Farah A, Salad A, Gele S, Gele A. Constraints to maternal healthcare access among pastoral communities in the Darussalam area of Mudug region, Somalia "a qualitative study". Frontiers in Public Health. 2023;11:1210401.
14. Chham S, Radovich E, Buffel V, Ir P, Wouters E. Determinants of the continuum of maternal health care in Cambodia: an analysis of the Cambodia demographic health survey 2014. BMC pregnancy and childbirth. 2021;21(1):410.
15. Muhirwe, L.B., Aagard, M. Completion of four or more ANC visits among women of reproductive age in Iganga district in Uganda: a quantitative study on the role of service-level factors. BMC Health Serv Res 23, 906 (2023). https://doi.org/10.1186/s12913-023-09913-7.
16. Muchie, K.F. Quality of antenatal care services and completion of four or more antenatal care visits in Ethiopia: a finding based on a demographic and health survey. BMC Pregnancy Childbirth 17, 300 (2017). https://doi.org/10.1186/s12884-017-1488-0.
17. Jawad M, Hone T, Vamos EP, Cetorelli V, Millett C. Implications of armed conflict for maternal and child health: A regression analysis of data from 181 countries for 2000–2019. PLoS Medicine. 2021;18(9):e1003810.
18. Ronan K, Jenkins M. Somalia: Overview of corruption and anti-corruption. Berlin: collaboration between U4 and Transparency. 2017.
19. Vian T. Anti-corruption, transparency and accountability in health: concepts, frameworks, and approaches. Global health action. 2020;13(sup1):1694744.
20. Factor R, Kang M. Corruption and population health outcomes: an analysis of data from 133 countries using structural equation modeling. International journal of public health. 2015;60:633-41.
21. Hanf M, Van-Melle A, Fraisse F, Roger A, Carme B, Nacher M. Corruption kills: estimating the global impact of corruption on children deaths. PloS one. 2011;6(11):e26990.
22. Mehboob R, Gilani SA, Khalid S, Hassan A, Alwazzan A. Maternal mortality ratio in low income developing countries. Global women's health. 2021;10.
23. Nishimwe A, Ibisomi L, Nyssen M, Conco DN. The effect of a decision-support mHealth application on maternal and neonatal outcomes in two district hospitals in Rwanda: pre–post intervention study. BMC Pregnancy and Childbirth. 2022;22(1):52.
24. Asratie MH, Muche AA, Geremew AB. Completion of maternity continuum of care among women in the post-partum period: Magnitude and associated factors in the northwest, Ethiopia. PloS one. 2020;15(8):e0237980.
25. Tessema ZT, Yazachew L, Tesema GA, Teshale AB. Determinants of postnatal care utilization in sub-Saharan Africa: a meta and multilevel analysis of data from 36 sub-Saharan countries. Italian journal of pediatrics. 2020;46:1-11.

26. Dadi TL, Medhin G, Kasaye HK, Kassie GM, Jebena MG, Gobezie WA, et al. Continuum of maternity care among rural women in Ethiopia: does place and frequency of antenatal care visit matter? Reproductive health. 2021;18:1-12.
27. Samuel O, Zewotir T, North D. Decomposing the urban–rural inequalities in the utilisation of maternal health care services: evidence from 27 selected countries in Sub-Saharan Africa. Reproductive Health. 2021;18:1-12.

Figure Click here to access/download;Figure;Figures-CoC.tiff

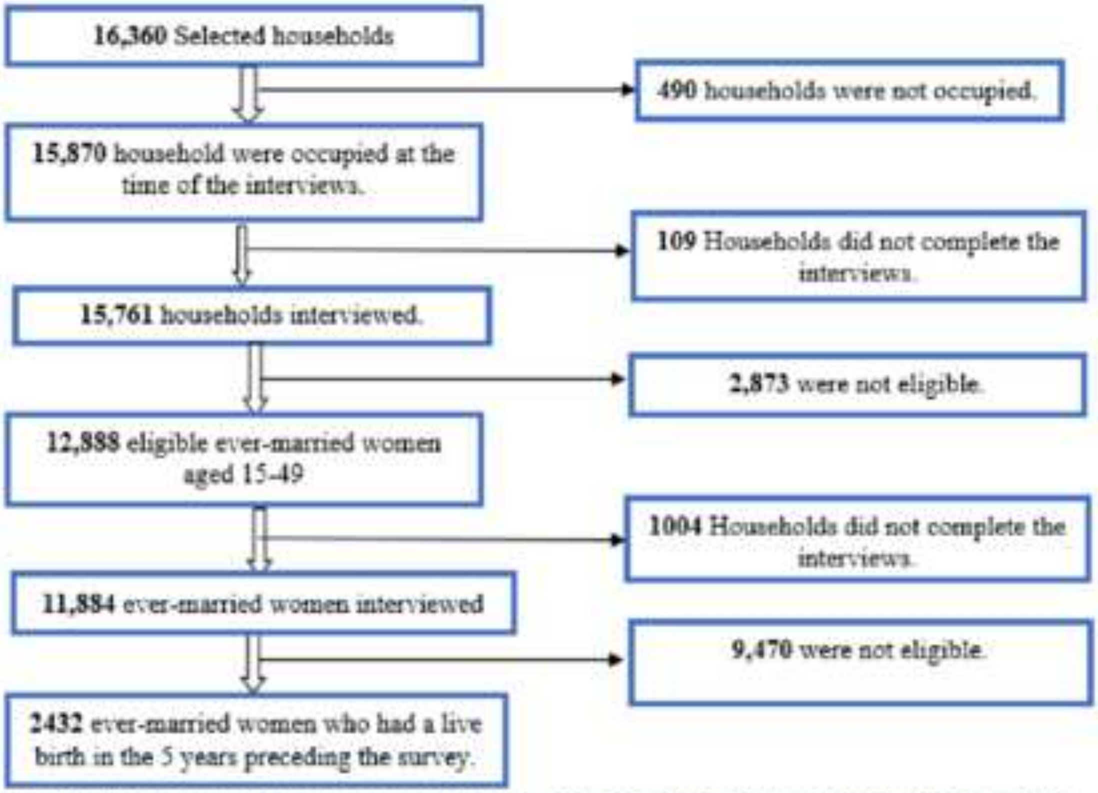

Figure 1: selection process of eligible women aged 15-49 years old using Somalia Health and Demographic Survey 2020.

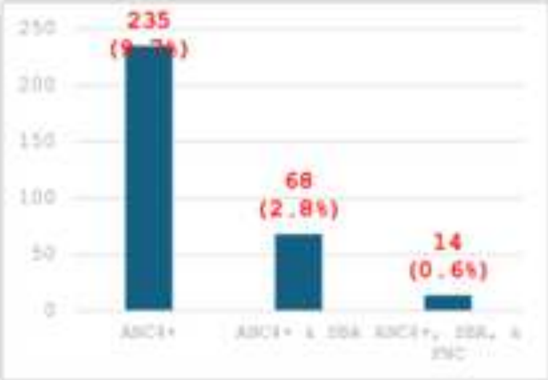

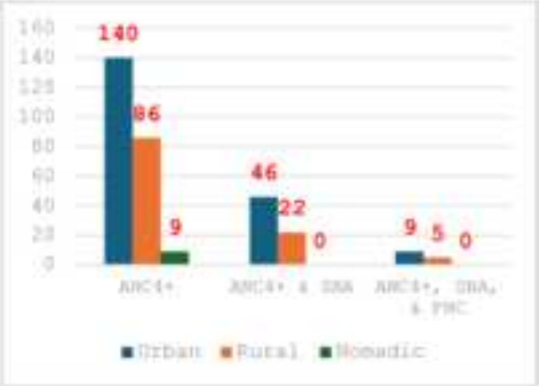

Figure 1: Continuum of maternal healthcare
residence

Figure 2: Continuum of maternal healthcare by

Click here to access/download
Supporting Information
SOIR71SV.sav

Level of Completion of Maternity Continuum of Care among ever-married women: An analysis of Somalia Health and Demographic Survey 2020

Adam A. Mohamed, Ayşe Akın, Abdi Gele

Abstract

Introduction: Somalia is continuing its retrieval from three decades of underdevelopment, political instability, civil unrest, and protracted humanitarian crises. Somalia has one of the worst maternal conditions in the world. For instance, the maternal mortality ratio is 692 per 100,000 live births. Extra efforts are needed to improve maternal health. In this study, we aim to investigate the level of completion and coverage along maternity continuum of care in Somalia

Method: The study used data from Somalia Health and Demographic Survey 2020. We restricted our analysis to ever-married women who had a live birth in the five years preceding the survey (n = 2432). Completion of the continuum of maternity care was the outcome variable for this study. It was constructed into a binary variable with complete coded as 1 and incomplete coded as 0. We categorized it into three models: ANC4+ as the first model, ANC4+ & SBA as the second model, and ANC4+ & SBA & PNC as the third model.

Results: More than half of the women (53.1%) had their most recent births at the age ≤ 19 years old. of all the mothers (n=2432), only 235 (9.7%) had at least four or more of the recommended antenatal care (ANC4+), 68 (2.8%) of them utilized skilled birth attendants and only 14 (0.6%) of women had received all of the three maternal healthcare services (ANC4+, SBA, and PNC with in 48hrs). About 78.1% of the mothers did not attend any of the three CoC services.

Conclusion: Maternal health care utilization decreases as they progress from ANC4+ to PNC utilization. The government and partners should design and implement strategies to improve maternal healthcare utilization specific to rural and nomads, less educated, not working, low income, and less power to decision-making.

Keywords: Continuum of care, Antenatal Care, Skilled birth attendant, Postnatal care, Somalia

1. Background

Sub-Saharan Africa (SSA) has the highest rates of maternal mortality in the world, accounting almost 90% of the global burden of maternal mortalities (1, 2). This high burden of maternal mortality in the region has been attributed to preventable factors such as low antenatal care (ANC) utilization, low uptake of skilled attendant delivery and postnatal care (PNC) (3). In 2020, the MMR in the African Region was 531 deaths per 100 000 live births, accounted for 69% of global maternal deaths in 2020 (4). From 2000 to 2020 the maternal mortality ratio dropped by about 34% worldwide. However, in many low- and middle-income countries maternal mortality remains a significant public health problem with nearly 94% of all maternal deaths occurring in low resource settings to date (5).

Somalia is continuing its retrieval from three decades of underdevelopment, political instability, civil unrest, and protracted humanitarian crises. The country has suffered multiple emergencies including flooding, drought, famine, locust attacks, other climate change shocks which resulted in many deaths and large-scale population displacements (6). Somalia has a population of 17 million, with 44 percent of the population living in urban areas, 23 percent living in rural areas, 26 percent in nomadic areas, and 9 percent living in Internally Displaced settings (IDPs) (7). The country has one of the weakest health care systems in Sub-Saharan Africa, due to limited state capacity, poverty-related deprivation, corruption, and longstanding armed conflicts. Therefore, Somalia has some of the lowest health indicators in the world (6). The fragile health system in Somalia is shaped by various administrations that adopted different policies, priorities, and health care service approaches, often influenced by local state administrations and international paradigms and resolutions (8).

Somalia is among the 15 countries that WHO marked as very high alert countries for maternal, newborn, and under 5 deaths. Most of the causes are either preventable or treatable. Somalia has one of the worst maternal conditions in the world. For instance, the maternal mortality ratio is 692 per 100,000 live births. Poor maternal health care delivery in the rural communities results the majority of the maternal, newborn, and child deaths during pregnancy, childbirth, and after delivery in Somalia (9). This is due to the unavailability and low utilization of maternal healthcare services including skilled birth attendance, emergency obstetric care, postnatal care, and family planning. According to a predictive analysis on the trends of maternal mortality ratio, Somalia will not meet the sustainable development goal target 3.1 of reducing the maternal mortality ratio to

less than 70 per 100,000 live births. The country will not also meet the targets in reducing neonatal mortality to below 12 per 1000 live births and under-5 mortality to below 25 per 1000 live births by 2030 (10). According to Somalia's Voluntary National Reviews Report 2022 and voluntary national reviews in East African countries on Sustainable Development Goals specially goal 3, Somalia still faces significant challenges in maternal mortality rates, which can be attributed to low uptake of antenatal care and postnatal care and a low number of deliveries at health facilities or with skilled health care providers. Somalia is unlikely to achieve the SDGs given the lack of evidence-based knowledge that inform government interventions to improve maternal and newborn health (11).

Continuum of Care (CoC) refers to the continuity of care throughout pregnancy, birth, and after delivery (i.e., use of antenatal care, skilled birth attendance, and postnatal care). Ensuring that continuity of care for maternal, newborn, and child health has become a key for improving the health of the mothers, newborns, and children. The continuum of care has newly been emphasized as a core principle of programmes for maternal, newborn, and child health, and as a method to reduce the burden of the maternal, newborn, and child deaths (12).

The main services for continuum of care like antenatal care, facility delivery, and postnatal care are recognized to reduce maternal and child mortality and morbidity in high burden settings. Maternal and child mortality rates are indicators that are used globally to determine the health, economy, and developmental status of countries. Although the Somalia government has taken a significant step to increase the availability and access of the maternal care, still a low proportion of mothers utilize the services specially the urban areas (13). Improving the utilization of the maternity continuum of care relies on a better understanding of the barriers and gaps affecting the uptake to each service i.e., ANC4+, SBA, and PNC. In this study, we aim to investigate the level of completion and coverage along maternity continuum of care in Somalia.

2. Methods

2.1. Data source and study participants

This study used data from the 2020 Somalia Health and Demographic Survey which is a nationally representative household survey that was designed to collect, analyze, and disseminate demographic data on reproductive health, maternal and child mortality, family planning and fertilities, nutrition, and health care utilization in Somalia. The main objective of the Somali Health and Demographic Survey was to provide evidence on the health and demographic characteristics

of the Somali population that will guide the development of programmes and formulation of effective policies. This nationally representative survey used a multistage cluster sampling design to collect data on reproductive health, maternal and child mortality, family planning and fertilities, nutrition, and health utilization in Somalia. About 11,884 ever-married women of reproductive age (15-49~~)~~ completed the interviews. Our study focused on ever-married women who had a live birth in the five years preceding the survey (n=2432) as you can see in figure 1 below. The survey further restricted the women's most recent live births in the recall period. 9,470 were not eligible due to either not having children less than 5 years or incomplete data in the maternal and child health questions or not fitting the targets and the analysis removed them. -Marriage is associated with childbearing and women can have a child only if she is married as accepted in the community. That is why we restricted our study on only the ever-married women.

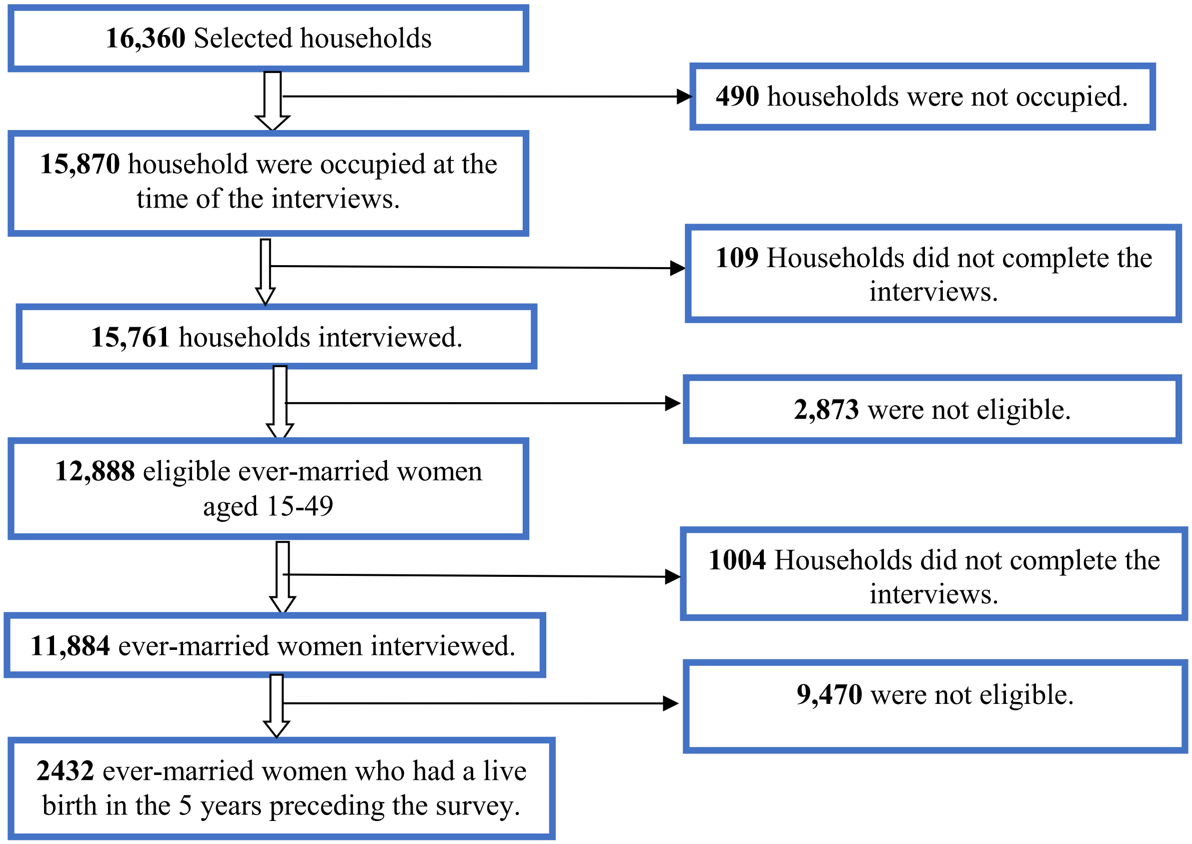

Figure 1: selection process of eligible women aged 15-49 years old using Somalia Health and Demographic Survey 2020.

~~**2.2.Study Variables**~~

~~**2.2.1. Dependent Variables**~~

The maternity continuum of care in this study composed of three dummy variables as dependent variables in the study: ANC4+, SBA, and PNC within 48hours. We defined the maternity continuum of care as the completion and continuity of care throughout pregnancy, birth, and after delivery (i.e., use of antenatal care, skilled birth attendance, and postnatal care). We defined ANC4+ percentage of women with a live birth who received WHO-recommended at least four times antenatal care visits over the course of their pregnancy. We defined Skilled Birth Attendant (SBA) as births attended by a skilled health personnel such as midwife, doctor, nurse, or other health care professional in a given period. We defined postnatal care as the care women and her newborn baby received immediately after the birth and we limited to our continuum of care within two days or 48 hours after childbirth. We constructed into binary variables to each of the three dependent variables with complete coded as 1 and incomplete coded as 0. Continuum of Maternity care was assumed complete if the mother utilized all of the three services like receiving at least four ANC services, delivered by a skilled birth attendant, and received postnatal check within 48 hours after delivery during the most recent pregnancy.

2.2.2. Independent Variables

We included three predictors in the models such (1) socio-demographic factors (2) socio-economic factors and (3) cultural factors which were all found in the previous literatures. Socio-demographic factors included maternal age at the recent births, women's level of education, marital status, birth order of the most recent child, and number of children. Socio-economic factors included place of residence (urban, rural, or nomadic), women's employment (working, or not working, household wealth quintile (poorest, poorer, middle, richer, or richest). Cultural factors included exposure to mass media (listing to radio as yes or no). We categorized a woman as having access to mass media if she had access to radio at least once a week and no access if she did not listen to a radio at least once a week. We assessed women's empowerment or decision-making power in determining own healthcare and we scored full empowerment for decisions involving the woman or jointly with her husband (full power) and decision taken without the woman's involvement (no power) (14).

2.3.2.2. Data analysis

We used STATA software (version 18) and sampling weight for all analysis with survey data analysis command (svy) to account for the cluster survey design and missing responses. The study did not proportionally sample on urban, rural, and nomadic domains hence urban overrepresentation may occur. Large part of the population refused to finalize the questionnaires

specially the maternal and child health section, hence causing incomplete interviews. The SHDS data have a missing information due to some aspects of the population were not covered. The SHDS data in Somalia was not meant to tract detailed aspects of maternity care which is making difficult to analyze the maternity continuum of care. We checked data for completeness and then cleared. We then conducted a descriptive analysis (number, frequency, and percentage) to summarize the characteristics of the study population and each variable. We assessed the level of coverage for each maternal health service (ANC, SBA, and PNC) separately and then together as a combined continuum of maternity care. Variance Inflation Factor was used to test the presence of collinearity between the independent variables using STATA 18.

3. Results

3.1.Characteristics of the study participants

Table 1 summarizes the background characteristics of ever-married mothers included in this study. The mean age of the women in the study was 22.8 years (SD = 4.2) with more than half of the women (53.1%) had their most recent births at the age of ≤19 years while 90.2% of the women in the study were married during the survey period. Above sixty percent of the women were living in urban area and seventy-seven had no education. 96.1% of the women were not working, 74.5% of them had 1-2 children, and 90.6% had not accessed to mass media (radio exposure). Wealth quintile distribution among the households were almost homogenous proportion with the highest being the poorest (25.5%) and the lowest being the middle group (16.7%) as you can see the below table 1.

Table 1: Socio-demographic characteristics of ever-married women who had at least one a live birth in the five years preceding the survey (Weighted sample size = 2432 & unweighted sample size = 2414)

Variable	Categories	Weighted Number (2432)	Percentage (%)
**Maternal age at birth (years)**	<20	1,291	53.1
	20-34	1,125	46.3
	35-49	16	0.6
**Birth Order**	1	906	37.3
	2	908	37.3
	3	525	21.6
	4 or more	93	3.8
**Residence**	Urban	1,475	60.6
	Rural	653	26.9
	Nomadic	304	12.5
**Marital Status**	Married	2,193	90.2
	Divorced/widowed	239	9.8

**Mother's Education**	No Education	1,872	77.0
	Primary	370	15.2
	Secondary & above	190	7.8
**Mother's Employment**	Working	96	3.9
	Not working	2,336	96.1
**Number of Children**	1-2	1,813	74.5
	3-4	598	24.6
	>=5	21	0.9
**Decision-making power in determining own healthcare**	Women or jointly	1,285	52.8
	Husband or others	1,147	47.2
**Exposure to Radio**	Yes	229	9.4
	No	2,203	90.6
**Wealth Quintile**	Poorest	621	25.5
	Poorer	486	20.0
	Middle	406	16.7
	Richer	454	18.7
	Richest	465	19.1

3.2.Overall use of maternal health services in Somalia

Table 2 shows the descriptive analysis indicating that Somalia did not achieve a good antenatal care, institutional delivery, and postnatal care coverage. Around **two-third** of the study participants (66.2%) did not receive antenatal care visits, and only 24.2% received ANC between 1-3 visits whereas 9.7% of the women in the study attended the recommended four or more antenatal care (ANC4+) visits. Less than 14% of the deliveries was attended by skilled provider. For postnatal care, only 2.7% of the women had a PNC check within 48 hours after delivery regardless of their delivery place.

Characteristics	Categories	Weighted Number (2432)	Percentage (%)
**ANC visits**	No ANC	1,608	66.2
	1-3ANC	589	24.2
	ANC4+	235	9.7
**Delivered by SBA**	Skilled provider	327	13.4
	Unskilled provider	1,478	60.8
	No one	47	1.9
	Don't know/missing	580	23.8
**PNC visits**	First 48hrs	67	2.7
	More than 48hrs	2	0.1
	No PNC/Don't know	2,363	97.2

Table 2: Number and Percentage of mothers and their use of maternal health services.

Maternity Continuum of Care: of all the mothers (n=2432), only 235 (9.7%) had at least four or more of the recommended antenatal care (ANC4+) visits during their recent pregnancy and among this, only 9 mothers live in nomadic settings. Out of the women who have received four or more

ANC care, only 68 (2.8%) of them utilized skilled birth attendants in their most recent pregnancies. Regarding continuum of maternal health services, only 14 (0.6%) of women had received all of the three maternal healthcare services (ANC4+, SBA, and PNC with in 48hrs).

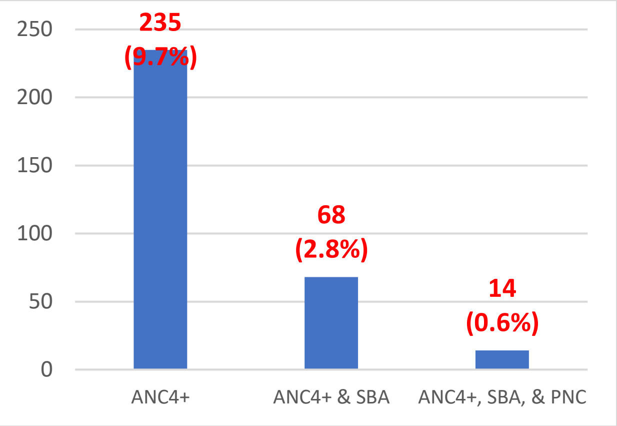

Figure 2: Continuum of maternal healthcare

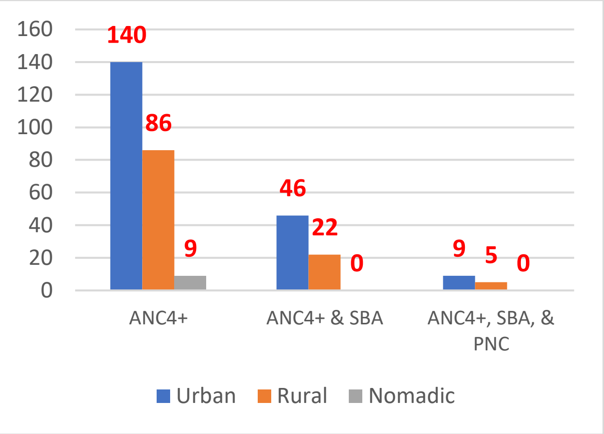

Figure 3: Continuum of maternal healthcare by residence

1.1.Pathways of maternal healthcare use

Considering the study's three main outcomes (ANC4+, SBA, and PNC), we created eight different pathways or combinations of maternal health service utilization i.e., from not receiving any of the three maternal health services to complete use of all three maternal health services (Table 3). More than two-third of the mothers in the study (78.1%) did not receive any of the three maternal health services as shown in pathway 1. Notably, only 14 mothers (0.6%) received all of the three maternal health services along the continuum (pathway 8). Three groups that attended any two of the three maternal health services (pathway 3, 6, and 7) accounted for 2.2%, 0.5%, and 0.1% respectively. Lastly, the three groups that utilized only one of the three maternal services (pathways 2, 4, and 5) accounted 6.7%, 1.6%, and 10.2% respectively.

Pathway	ANC4+	SBA	PNC within 48hrs	Frequency (Weighted Number)	Percentage
1	–	–	–	1,899	78.1
2	+	–	–	164	6.7
3	+	+	–	54	2.2
4	–	–	+	39	1.6
5	–	+	–	248	10.2
6	–	+	+	11	0.5
7	+	–	+	3	0.1
8	+	+	+	14	0.6
**Total**				**2,432**	**100%**

*Table 3: Percentage distribution of the three maternal health services received by women. **+** Received the service; **-** Did not receive the service.*

The table 3 above shows the combination of maternal health services (three major services) that mothers either received or did not receive. It is summarizing the three major outcome variables of the study i.e., antenatal care of four times or above, skilled birth attendant, post-natal care within 48 hours after delivery.

2. Discussion

This study assessed maternity continuum of care for the first time in Somalia. Access and use of maternity care services during pregnancy, childbirth, and the postnatal period from skilled providers are essential for the survival and wellbeing of the mother and newborn. It is particularly critical in setting where teenage pregnancy is common such as Somalia. Our study states that 53% of women gave birth their first child at the age of ≤ 19, while the completion of ANC4 was only 9.7%. This rate is much higher than the completion of ANC4 which is 59.9% in Uganda (15), and that of Ethiopia which is 33% (16). Protracted conflicts and instability may explain why women in Somalia have low prevalence of ANC4. Armed conflict has been described as an important contributor to persistent excess maternal and child deaths while it can severely reduce access to maternal health services and thus lead to poor maternal health outcomes (17). The effect of the three decades of conflict on the access to health care in Somalia is exacerbated by the pervasive corruption in the health sector, given the fact that systemic corruption and the large-scale misappropriation of state funds is the norm in Somalia (18). Somalia, like many other countries, corruption at the health sector range from smaller-scale acts by doctors and nurses who charge "informal payments" to larger-scale acts at the ministerial level, when people in power employ incompetent relatives or redirect resources away from those who need it for their own benefit (19). Hence, countries with high levels of corruption spend less on health care (20). In addition, high levels of corruption correlate with higher infant and child mortality rates (21). To improve maternal health care in Somalia, requires political and economic commitment, and genuine leadership is critical in achieving this agenda.

Our study shows that only 13.4% of women in Somalia gave birth with the help of skilled health provider. A prior study on 29 Sub-Saharan African countries found an average proportion of women who had skilled assistance during delivery was 75.3%, ranging from 38.4% in Chad to 93.7% in Rwanda (3). Skilled attendants have positive contribution in the reduction of maternal and newborn mortality and morbidity. A 40% decline in maternal deaths in SSA occurred between

2000 and 2017 was attributed, by WHO, to utilizing skilled birth attendant at delivery (22). The fact that only 13.4% of women in Somalia are using the services of skilled birth attendant, indicated that although utilization of the services of SBAs in SSA appears high, it is extremely low in Somalia. The SBA prevalence in Somalia can be improved by adopting prevailing successful interventions of countries with SBA successes such as Rwanda, whilst ~~taking into account~~considering of contextual variations (23). Our study finds that the overall completion of maternity continuum of care is less than 1%. This is much lower than that reported in Gondor Ethiopia 47% (24). This could be due to the efforts made by the Ethiopian government to in the recent years to strengthen maternity health care access.

Our study found that only 2.8% of Somali women have received postnatal care. Receiving postnatal care after delivery is vital for women and the baby due to the fact that over 65% of maternal and neonatal deaths occur during the first 42 days of postpartum and during the first 7 days of life respectively. A study on postnatal care utilization in 36 sub-Saharan Africa countries found a prevalence of PNC of 52.48% [95% CI: 52.33, 52.63], ranging from 73.51% in Central Africa region to 31.71% in Eastern Africa Region (25). A prior study found an association of armed conflicts with an increase of 36.9 maternal deaths per 100,000 live births, an increase of 2.8 infant (under 1 year old) deaths per 1,000 live births (17).

The continuity of maternity health care services is the care that a woman uses the three recommended cares of antenatal care (ANC), skill birth attendant (SBA), and postnatal care (PNC). Our study shows that only 0.6% of the women in Somalia have completed the three services; ANC4, SBA and PNC, while only 2.8% have completed ANC4 and SBA. The prevalence of maternal continuum of care in Somalia which is stated by our study (0.6%), is far much lower than the ~~prevalence~~ frequence of continuum of care in Ethiopia (47%) (24)~~Sub Saharan Africa (25.0%), which varied from 17.9.0% in East Africa to 7,418 (51.5% in Southern Africa (26). It is also much lower than the 35.81% pooled prevalence of completion of the maternity continuum of care among 33 sub Saharan African countries (27), it~~ and is even over 15 times lower than the continuum of care of rural women in Ethiopia (13%) (28). Further, our study shows a significant inequity in continuum of care by residence with zero continuum of care among women in pastoral communities that constitute 26% of total population in Somalia (7). A recent study in Somalia

stated that pastoralist women have limited access to maternal health care which supports our findings (13). The inequity between urban and rural women, in utilizing maternal health care services, is prevalent across Africa. A prior study of 27 African countries showed a prevalence of ANC utilization of 34.7% among urban women and 22.4% among in rural areas, while SBA prevalence was nearly 90% and 69% in urban women and rural women respectively (29).

This study has both strength and limitations. The main strength of this study is the use of weighted nationally representative data with a large sample, which makes it representative at national level. The SHDS data used a multi-stage cluster sampling design, and some groups or demographic categories might be over sampled hence, we weighed the SHDS data during analysis.

The current SDHS did not collect information on type of provider for each visit, therefore, we couldn't determine the type of provider for each visit. Further, approximately 2.6 million Somalis are currently displaced within their own country and the SHDS 2020 did not include the IDP domain in the data.

Conclusion.

The prevalence of completion of the maternity continuum of care was found to be lower than any country in Africa. Maternal health care utilization decreases as they progress from ANC4+ to PNC utilization. Inequity in the continuum of care is a chronic problem in Somalia where pastoralist women who constitute 26% of the population have zero completion of continuum of care. Health inequalities are avoidable because they are rooted in political and social decisions. Political and economic commitment is required in improving the access to maternal health care in the country. The findings of our study indicate that if the government does not target the rural and pastoralist communities, women's retention to the health services if existing would be minimum.

To reduce health inequalities between urban and pastoralists in Somalia, we need to act across a range of health-policy areas including policies to improve access to maternal health care in pastoralists setting in the country. The government and partners should design and implement strategies to improve maternal healthcare utilization specific to rural and nomads, less educated, not working, low income, and less power to decision-making. The government of Somalia should invest and increase the number of certified skilled birth attendants i.e., nurses, midwives, and obstetricians to provide safe and competent care during childbirth. To attract and retain skilled birth attendants in remote and rural areas, the government and its partners should implement incentive programs designed to the professional and certified midwives and obstetricians and

should train and deploy community health extension workers in rural areas to provide basic maternal health educations, identify pregnant women, and support and encourage them to seek health care.

Uncategorized References

1. Yaya S, Anjorin SS, Adedini SA. Disparities in pregnancy-related deaths: spatial and Bayesian network analyses of maternal mortality ratio in 54 African countries. BMJ global health. 2021;6(2):e004233.
2. Onambele L, Ortega-Leon W, Guillen-Aguinaga S, Forjaz MJ, Yoseph A, Guillen-Aguinaga L, et al. Maternal mortality in Africa: Regional trends (2000–2017). International journal of environmental research and public health. 2022;19(20):13146.
3. Budu E, Chattu VK, Ahinkorah BO, Seidu A-A, Mohammed A, Tetteh JK, et al. Early age at first childbirth and skilled birth attendance during delivery among young women in sub-Saharan Africa. BMC pregnancy and childbirth. 2021;21:1-12.
4. Ward ZJ, Atun R, King G, Dmello BS, Goldie SJ. Global maternal mortality projections by urban/rural location and education level: a simulation-based analysis. Eclinicalmedicine. 2024;72.
5. Organization WH. Trends in maternal mortality 2000 to 2020: estimates by WHO, UNICEF, UNFPA, World Bank Group and UNDESA/Population Division: World Health Organization; 2023.
6. Mackey KP, Kiptum SE. Health systems strengthening in fragile contexts: A partnership model in South West State, Somalia. Field Exchange 57. 2018:89.
7. Federal Republic of Somalia. POPULATION ESTIMATION SURVEY 2014 SOMALIA FOR THE 18 PRE-WAR REGIONS OF SOMALIA. 2014. chrome-extension://efaidnbmnnnibpcajpcglclefindmkaj/https://somalia.unfpa.org/sites/default/files/pub-pdf/Population-Estimation-Survey-of-Somalia-PESS-2013-2014.pdf.
8. Warsame AA. Somalia's Healthcare System: A baseline study & human capital development strategy. Heritage Institute and City University of Mogadishu. 2020.
9. Aden JA, Ahmed HJ, Östergren P-O. Causes and contributing factors of maternal mortality in Bosaso District of Somalia. A retrospective study of 30 cases using a Verbal Autopsy approach. Global health action. 2019;12(1):1672314.
10. Morrison J, Malik SMMR. Population health trends and disease profile in Somalia 1990–2019, and projection to 2030: will the country achieve sustainable development goals 2 and 3? BMC Public Health. 2023;23(1):1-9.
11. Chisika SN, Yeom C. Voluntary National Reviews on Implementation of Sustainable Development Goals: Key Lessons from East African Member States. SAGE Open. 2024;14(2):21582440241257319.
12. Kerber KJ, de Graft-Johnson JE, Bhutta ZA, Okong P, Starrs A, Lawn JE. Continuum of care for maternal, newborn, and child health: from slogan to service delivery. Lancet. 2007;370(9595):1358-69.
13. Duale HA, Farah A, Salad A, Gele S, Gele A. Constraints to maternal healthcare access among pastoral communities in the Darussalam area of Mudug region, Somalia "a qualitative study". Frontiers in Public Health. 2023;11:1210401.

14. Chham S, Radovich E, Buffel V, Ir P, Wouters E. Determinants of the continuum of maternal health care in Cambodia: an analysis of the Cambodia demographic health survey 2014. BMC pregnancy and childbirth. 2021;21(1):410.
15. Muhirwe, L.B., Aagard, M. Completion of four or more ANC visits among women of reproductive age in Iganga district in Uganda: a quantitative study on the role of service-level factors. BMC Health Serv Res 23, 906 (2023). https://doi.org/10.1186/s12913-023-09913-7.
16. Muchie, K.F. Quality of antenatal care services and completion of four or more antenatal care visits in Ethiopia: a finding based on a demographic and health survey. BMC Pregnancy Childbirth 17, 300 (2017). https://doi.org/10.1186/s12884-017-1488-0.
17. Jawad M, Hone T, Vamos EP, Cetorelli V, Millett C. Implications of armed conflict for maternal and child health: A regression analysis of data from 181 countries for 2000–2019. PLoS Medicine. 2021;18(9):e1003810.
18. Ronan K, Jenkins M. Somalia: Overview of corruption and anti-corruption. Berlin: collaboration between U4 and Transparency. 2017.
19. Vian T. Anti-corruption, transparency and accountability in health: concepts, frameworks, and approaches. Global health action. 2020;13(sup1):1694744.
20. Factor R, Kang M. Corruption and population health outcomes: an analysis of data from 133 countries using structural equation modeling. International journal of public health. 2015;60:633-41.
21. Hanf M, Van-Melle A, Fraisse F, Roger A, Carme B, Nacher M. Corruption kills: estimating the global impact of corruption on children deaths. PloS one. 2011;6(11):e26990.
22. Mehboob R, Gilani SA, Khalid S, Hassan A, Alwazzan A. Maternal mortality ratio in low income developing countries. Global women's health. 2021;10.
23. Nishimwe A, Ibisomi L, Nyssen M, Conco DN. The effect of a decision-support mHealth application on maternal and neonatal outcomes in two district hospitals in Rwanda: pre–post intervention study. BMC Pregnancy and Childbirth. 2022;22(1):52.
24. Asratie MH, Muche AA, Geremew AB. Completion of maternity continuum of care among women in the post-partum period: Magnitude and associated factors in the northwest, Ethiopia. PloS one. 2020;15(8):e0237980.
25. Tessema ZT, Yazachew L, Tesema GA, Teshale AB. Determinants of postnatal care utilization in sub-Saharan Africa: a meta and multilevel analysis of data from 36 sub-Saharan countries. Italian journal of pediatrics. 2020;46:1-11.
26. Alem, A.Z., Shitu, K. & Alamneh, T.S. Coverage and factors associated with completion of continuum of care for maternal health in sub-Saharan Africa: a multicountry analysis. BMC Pregnancy Childbirth 22, 422 (2022). https://doi.org/10.1186/s12884-022-04757-1.
27. Hunie Asratie M, Belay DG. Pooled prevalence and determinants of completion of maternity continuum of care in Sub-Saharan Africa: a multi-country analysis of recent demographic and health surveys. Frontiers in Global Women's Health. 2022;3:869552.
28. Dadi TL, Medhin G, Kasaye HK, Kassie GM, Jebena MG, Gobezie WA, et al. Continuum of maternity care among rural women in Ethiopia: does place and frequency of antenatal care visit matter? Reproductive health. 2021;18:1-12.
29. Samuel O, Zewotir T, North D. Decomposing the urban–rural inequalities in the utilisation of maternal health care services: evidence from 27 selected countries in Sub-Saharan Africa. Reproductive Health. 2021;18:1-12.

Review of Manuscript PGPH-D-24-00010

"Level of Completion of Maternity Continuum of Care among ever-married women: An analysis of Somalia Health and Demographic Survey 2020"

General Comments: The manuscript addresses a critical public health issue by assessing the completion levels of the maternity continuum of care among ever-married women in Somalia, utilizing data from the 2020 Somalia Health and Demographic Survey. This study is particularly relevant considering Somalia's high maternal mortality rate. The findings underscore significant gaps in maternal health service utilization, providing important insights for informing policy and programmatic interventions.

a. The focus on maternal health in a high-risk region like Somalia is important, given the country's significant health challenges and elevated maternal mortality rates.
b. The use of data from the 2020 Somalia Health and Demographic Survey strengthens the generalizability and relevance of the study's findings.
c. The manuscript is well-structured, providing a clear and logical progression through the study's objectives, methods, results, and conclusions.

Areas for Improvement

Methods and Literature Review:

1. The introduction could benefit from a more thorough review of existing literature on the maternity continuum of care, especially studies from similar contexts in Sub-Saharan Africa. This would better position the study within the broader research landscape and provide a clearer rationale.

Answer: This is address and LR improvement have been made.

2. While the manuscript references studies from other African countries, it could enhance its analysis by offering more detailed comparisons to better contextualize the findings.

Answer: We have added more detailed comparisons to better contextualize the findings

3. The use of descriptive analysis is appropriate, but including more detailed statistical methods, such as logistic regression, could help identify factors associated with the completion of the maternity continuum of care, offering a deeper understanding of service utilization determinants.

Answer: In this study as it stands is a descriptive and we did not intend to add determinants or correlations.

4. The manuscript lacks a clear explanation of how it addressed potential biases or limitations related to missing data or non-response. A more explicit discussion of these issues would strengthen the methodology section.

Answer: We have added a deep explanation of the missing data in the data analysis section.

5. The manuscript does not detail the weighting process or how weights were calculated. Providing a brief explanation of the weighting process would enhance readers' understanding of the representativeness and validity of the findings.

Answer: We have added a detailed weighting process importance in the strength and limitation paragraph.

6. A brief explanation of the rationale for restricting the analysis to "ever-married women could be helpful.

Answer: We have added an explanation of restricting the analysis to ever-married women in Somalia. Marriage is associated with childbearing and women can have a child only if she is married as accepted in the community. That is why we restricted our study on only the ever-married women.

Results, Discussion, and Conclusion

1. The results are clearly presented, but the manuscript could offer more detailed interpretations. For example, discussing the low completion rates of ANC4+, SBA, and PNC with insights into potential barriers and enablers would add depth.

Answer: More explanation of possible reasons for the low rate and completion rates of ANC4+, SBA, and PNC have been enriched to the manuscript.

2. The manuscript acknowledges the importance of addressing rural and nomadic populations but could expand on successful strategies from similar settings or propose tailored solutions for Somalia's unique context.

Answer: We have expanded the recommendation section and added examples from similar contexts. "The government and partners should design and implement strategies to improve maternal healthcare utilization specific to rural and nomads, less educated, not working, low income, and less power to decision-making. The government of Somalia should invest and increase the number of certified skilled birth attendants i.e., nurses, midwives, and obstetricians to provide safe and competent care during childbirth. To attract and retain skilled birth attendants in remote and rural areas, the government and its partners should implement incentive programs designed to the professional and certified midwives and obstetricians and should train and deploy community health extension workers in rural areas to provide basic maternal health educations, identify pregnant women, and support and encourage them to seek health care".

3. The discussion section reiterates the results but could benefit from a deeper analysis of the implications of these findings for policy and practice. For example, what specific policy changes or health system interventions are needed to improve the completion of the maternity continuum of care in Somalia?

Answer: We have enriched examples of policies and strategies in the manuscript.

4. The discussion on corruption's impact on health services is relevant, but providing concrete examples or data would make the argument more compelling.

Answer: Though we can not get a direct relationship, we have referenced other similar studies in the discussion section.

5. The tables and figures are generally clear, but the manuscript could benefit from an additional figure or flowchart that visualizes the pathways of care, helping readers to better understand the various combinations of service utilization.

Answer: The study has a clear table (3) showing 8 pathways for the completion of continuum of care.

6. The conclusion is concise but could be more specific in its recommendations, suggesting actionable steps for the Somali government, international donors, and NGOs working in the region.

Answer: We have expanded the conclusion section.

Minor Issues:

1. The citations in the main manuscript should be revised to align with PLOS Global Health referencing style.
Answer: We have aligned the citations with PLOS Global Health referencing style. We used the Vancouver referencing style.
2. There are minor grammatical errors throughout the manuscript. A thorough proofreading is recommended to enhance readability.
Answer: We have made a line-by-line proofreading.
3. The references are generally well-cited, but incorporating more recent studies on the maternity continuum of care and maternal health in conflict settings would strengthen the manuscript's academic rigor.
Answer: Added more recent studies in the manuscript.
4. Some references are inaccurately attributed. For example, the manuscript incorrectly states, "This rate is much higher than the completion of ANC4 in Uganda (59.9%) ...".
Answer: We have corrected the inaccurate figures and sentences.
5. The citations in the manuscript should be revised to comply with the PLOS Global Health referencing style.
Answer: We have revised the citations.

Recommendation

Minor Revisions: The manuscript is well-crafted and addresses a significant public health issue. However, it would benefit from the suggested revisions, particularly in enhancing the methodology, deepening the discussion, and expanding the literature review. I recommend that the authors implement these revisions to further strengthen the manuscript's contributions to the field.

Other reviews

Review Comments to the Author

Please use the space provided to explain your answers to the questions above. You may also include additional comments for the author, including concerns about dual publication, research ethics, or publication ethics. (Please upload your review as an attachment if it exceeds 20,000 characters).

Reviewer #1: The manuscript, as a descriptive study, addresses a critical public health issue in Somalia, and meets PLOS Global Health criteria for publication. However, given the richness of the available data, incorporating a more in-depth inferential analysis could provide valuable insights by identifying factors or predictors associated with the outcome (maternal continuum of care).

Answer: In this study as it stands is a descriptive and we did not intend to add determinants or correlations.

Reviewer #2: comments to the author

The authors pic an important topic in the field of public health entitled “Level of Completion of Maternity Continuum of Care among ever-married women: An analysis of Somalia Health and Demographic Survey 2020” particularly as the authors described in the country which has the highest maternal mortality in Sub-

Saharan Africa, Somalia. thank you for submitting such important and timely title for the journal. However, I have some comments and concerns to enhance the quality of this manuscript before publication.

General typographical comments

Abstract section: The sentence “This study used the first ever DHS data in Somalia and if not get any funding” needs attention and rewrite with correct spelling

Answer: This is considered and re-corrected.

it would have been better if the authors have been inserted page numbers and line numbers

Answer: This is considered, and page numbers inserted.

#Affiliations

Author 2 and author 3 should write their department first in the affiliation

Answer: Adam A. Mohamed[1,2*], Ayşe Akın[2], Sare Mihciokur[2], Sarp Üner[3], Abdi Gele[4]

[1] Department of Program Quality & Development, Save the Children, Mogadishu, Somalia

[2] Department of Public Health, Faculty of Health Sciences, Başkent University, Ankara, Turkey

[3] Department of Public Health, Faculty of Medicine, Lokman Hekim University, Ankara, Turkey

[4] Department of Research, Norwegian Institute of Public Health, Oslo, Norway

***Adam Abdulkadir Mohamed** Correspondence: Adamafrican@gmail.com

Methods: in your selection procedure it shows that “15,761 households interviewed” and “2,873 were not eligible.” After that it stated and showed that “12,888 eligible ever-married women aged 15-49” however, after interview “9,470 were not eligible” why? the authors shall clearly show the selection process for the scientific community it is confusing.

Answer: 9,470 were not eligible due to either not having children less than 5 years or incomplete data in the maternal and child health questions or not fitting the targets and the analysis removed them.

# In your Dependent and independent variables:

Why did the authors list or display the independent and dependent variables? Since you have already discussed and reported the descriptive statistics, why don't you analyze the associations—at the very least, the most basic one, the chi square association—in this case?

Describe the dependent variable and the metrics used to measure it. For instance, what were the metrics used to evaluate the prenatal, institutional delivery, and postnatal care for each? The authors limit postnatal care, in particular, to 48 hours after delivery; however, they do not specify the number of visits that should take place during this time. The authors should also provide an explanation for their decision to use 48 hours rather than the 42-day postpartum period and at least 4 PNC visits.

Answer: We used the 48 hours postpartum period since it is the critical period window which can emphasize the most urgent phase for maternal and newborn interventions. The data we have

are almost the same for both 48 hours and 42 days (low utilization of PNC services beyond 48 hours). We also compared our study with other studies that used 48 hours PNC visits.

# Discussion section

“A prior study on 29 Sub-Saharan African countries found an average proportion of women who had skilled assistance during delivery was 75.3%, ranging from 38.4% in Chad to 93.7% in Rwanda However, our study shows that only 13.4% of women in Somalia gave birth with the 10 help of skilled health provider.” The author attempts to compare their findings with those of previous studies. It would be preferable to compare with other comparable descriptive research because these studies are distinct from one another and cannot make sense when descriptive frequency-based results are compared to proportion-based findings.

Answer: We are comparing the descriptive figures from those countries like Chad (lowest) and Rwanda (highest) and we did not use the aggregate or average proportion figures. We have revisited this sentence too.

“The low utilization of SBA in Somalia can be improved by adopting prevailing successful interventions of countries with SBA successes such as Rwanda, whilst taking into account of contextual variations” needs reference citation

Answer: We have added the proper citations.

The authors stated that” The prevalence of maternal continuum of care in Somalia which is stated by our study (0.6%)” can the author put the confidence interval for this prevalence? I think it is a descriptive result and frequency not a “prevalence” furthermore, the authors stated that “The prevalence of maternal continuum of care in Somalia which is stated by our study (0.6%), is far much lower than the prevalence of continuum of care in Sub-Saharan Africa (25.0%), which varied from 17.9.0% in East Africa to 7,418 (51.5% in Southern Africa (27). It is also much lower than the 35.81% pooled prevalence of completion of the maternity continuum of care among 33 sub-Saharan African countries” It is not clear how to compare this one descriptive result with the pooled prevalence; instead, it would be appropriate to compare and discuss the studies with other comparable papers and setting outcomes.

Answer: We have revised this prevalence and proportions related figures and used similar papers.

I appreciate the authors' initiative and motivation, especially with regard to the crucial public health issue of the maternal health continuum of care in one of the areas with the highest rates of maternal
