# Supplementary material for: Level of completion of maternity continuum of care among ever-married women: An analysis of Somalia’s health and demographic survey 2020
Source: PLOS Glob Public Health. 2025 Jan 10;5(1):e0004102. doi: 10.1371/journal.pgph.0004102 (PMC11723541; doi:10.1371/journal.pgph.0004102)
Supplement: Attachment — Submitted filename: Response to Reviewers-Rebuttal.docx [file pgph.0004102.s003.docx]

**Review of Manuscript PGPH-D-24-00010**

**“**Level of Completion of Maternity Continuum of Care among ever-married women: An analysis of Somalia Health and Demographic Survey 2020”

**General Comments:** The manuscript addresses a critical public health issue by assessing the completion levels of the maternity continuum of care among ever-married women in Somalia, utilizing data from the 2020 Somalia Health and Demographic Survey. This study is particularly relevant considering Somalia's high maternal mortality rate. The findings underscore significant gaps in maternal health service utilization, providing important insights for informing policy and programmatic interventions.

a. The focus on maternal health in a high-risk region like Somalia is important, given the country's significant health challenges and elevated maternal mortality rates.
b. The use of data from the 2020 Somalia Health and Demographic Survey strengthens the generalizability and relevance of the study's findings.
c. The manuscript is well-structured, providing a clear and logical progression through the study’s objectives, methods, results, and conclusions.

**Areas for Improvement**

**Methods and Literature Review:**

1. The introduction could benefit from a more thorough review of existing literature on the maternity continuum of care, especially studies from similar contexts in Sub-Saharan Africa. This would better position the study within the broader research landscape and provide a clearer rationale.

**Answer:** This is address and LR improvement have been made.

2. While the manuscript references studies from other African countries, it could enhance its analysis by offering more detailed comparisons to better contextualize the findings.

**Answer:** We have added more detailed comparisons to better contextualize the findings

3. The use of descriptive analysis is appropriate, but including more detailed statistical methods, such as logistic regression, could help identify factors associated with the completion of the maternity continuum of care, offering a deeper understanding of service utilization determinants.

**Answer:** In this study as it stands is a descriptive and we did not intend to add determinants or correlations.

4. The manuscript lacks a clear explanation of how it addressed potential biases or limitations related to missing data or non-response. A more explicit discussion of these issues would strengthen the methodology section.

**Answer:** We have added a deep explanation of the missing data in the data analysis section.

5. The manuscript does not detail the weighting process or how weights were calculated. Providing a brief explanation of the weighting process would enhance readers' understanding of the representativeness and validity of the findings.

**Answer:** We have added a detailed weighting process importance in the strength and limitation paragraph.

6. A brief explanation of the rationale for restricting the analysis to “ever-married women could be helpful.

**Answer:** We have added an explanation of restricting the analysis to ever-married women in Somalia. Marriage is associated with childbearing and women can have a child only if she is married as accepted in the community. That is why we restricted our study on only the ever-married women.

**Results, Discussion, and Conclusion**

1. The results are clearly presented, but the manuscript could offer more detailed interpretations. For example, discussing the low completion rates of ANC4+, SBA, and PNC with insights into potential barriers and enablers would add depth.

**Answer:** More explanation of possible reasons for the low rate and completion rates of ANC4+, SBA, and PNC have been enriched to the manuscript.

2. The manuscript acknowledges the importance of addressing rural and nomadic populations but could expand on successful strategies from similar settings or propose tailored solutions for Somalia’s unique context.

**Answer:** We have expanded the recommendation section and added examples from similar contexts. “The government and partners should design and implement strategies to improve maternal healthcare utilization specific to rural and nomads, less educated, not working, low income, and less power to decision-making. The government of Somalia should invest and increase the number of certified skilled birth attendants i.e., nurses, midwives, and obstetricians to provide safe and competent care during childbirth. To attract and retain skilled birth attendants in remote and rural areas, the government and its partners should implement incentive programs designed to the professional and certified midwives and obstetricians and should train and deploy community health extension workers in rural areas to provide basic maternal health educations, identify pregnant women, and support and encourage them to seek health care”.

3. The discussion section reiterates the results but could benefit from a deeper analysis of the implications of these findings for policy and practice. For example, what specific policy changes or health system interventions are needed to improve the completion of the maternity continuum of care in Somalia?

**Answer:** We have enriched examples of policies and strategies in the manuscript.

4. The discussion on corruption’s impact on health services is relevant, but providing concrete examples or data would make the argument more compelling.

**Answer:** Though we can not get a direct relationship, we have referenced other similar studies in the discussion section.

5. The tables and figures are generally clear, but the manuscript could benefit from an additional figure or flowchart that visualizes the pathways of care, helping readers to better understand the various combinations of service utilization.

**Answer:** The study has a clear table (3) showing 8 pathways for the completion of continuum of care.

6. The conclusion is concise but could be more specific in its recommendations, suggesting actionable steps for the Somali government, international donors, and NGOs working in the region.

**Answer:** We have expanded the conclusion section.

**Minor Issues:**

1. The citations in the main manuscript should be revised to align with PLOS Global Health referencing style.

**Answer:** We have aligned the citations with PLOS Global Health referencing style. We used the Vancouver referencing style.

2. There are minor grammatical errors throughout the manuscript. A thorough proofreading is recommended to enhance readability.

**Answer:** We have made a line-by-line proofreading.

3. The references are generally well-cited, but incorporating more recent studies on the maternity continuum of care and maternal health in conflict settings would strengthen the manuscript's academic rigor.

**Answer:** Added more recent studies in the manuscript.

4. Some references are inaccurately attributed. For example, the manuscript incorrectly states, "This rate is much higher than the completion of ANC4 in Uganda (59.9%) ...".

**Answer:** We have corrected the inaccurate figures and sentences.

5. The citations in the manuscript should be revised to comply with the PLOS Global Health referencing style.

**Answer:** We have revised the citations.

**Recommendation**

**Minor Revisions**: The manuscript is well-crafted and addresses a significant public health issue. However, it would benefit from the suggested revisions, particularly in enhancing the methodology, deepening the discussion, and expanding the literature review. I recommend that the authors implement these revisions to further strengthen the manuscript's contributions to the field.

**Other reviews**

**Review Comments to the Author**

Please use the space provided to explain your answers to the questions above. You may also include additional comments for the author, including concerns about dual publication, research ethics, or publication ethics. (Please upload your review as an attachment if it exceeds 20,000 characters).

Reviewer #1: The manuscript, as a descriptive study, addresses a critical public health issue in Somalia, and meets PLOS Global Health criteria for publication. However, given the richness of the available data, incorporating a more in-depth inferential analysis could provide valuable insights by identifying factors or predictors associated with the outcome (maternal continuum of care).

**Answer:** In this study as it stands is a descriptive and we did not intend to add determinants or correlations.

Reviewer #2: comments to the author
The authors pic an important topic in the field of public health entitled “Level of Completion of Maternity Continuum of Care among ever-married women: An analysis of Somalia Health and Demographic Survey 2020” particularly as the authors described in the country which has the highest maternal mortality in Sub-Saharan Africa, Somalia. thank you for submitting such important and timely title for the journal. However, I have some comments and concerns to enhance the quality of this manuscript before publication.
\# General typographical comments
\# Abstract section: The sentence “This study used the first ever DHS data in Somalia and if not get any funding” needs attention and rewrite with correct spelling

**Answer:** This is considered and re-corrected.
\# it would have been better if the authors have been inserted page numbers and line numbers

**Answer:** This is considered, and page numbers inserted.
**#Affiliations**
Author 2 and author 3 should write their department first in the affiliation

**Answer:** Adam A. Mohamed**^1,2^**^*^, Ayşe Akın**^2^,** Sare Mihciokur^2^, Sarp Üner**^3^,** Abdi Gele^4^

**^1^** Department of Program Quality & Development, Save the Children, Mogadishu, Somalia

**^2^** Department of Public Health, Faculty of Health Sciences, Başkent University, Ankara, Turkey

**^3^** Department of Public Health, Faculty of Medicine, Lokman Hekim University, Ankara, Turkey

**^4^** Department of Research, Norwegian Institute of Public Health, Oslo, Norway

***Adam Abdulkadir Mohamed** Correspondence: Adamafrican@gmail.com

\# Methods: in your selection procedure it shows that “15,761 households interviewed” and “2,873 were not eligible.” After that it stated and showed that “12,888 eligible ever-married women aged 15-49” however, after interview “9,470 were not eligible” why? the authors shall clearly show the selection process for the scientific community it is confusing.

**Answer:** 9,470 were not eligible due to either not having children less than 5 years or incomplete data in the maternal and child health questions or not fitting the targets and the analysis removed them.
**# In your Dependent and independent variables:**
Why did the authors list or display the independent and dependent variables? Since you have already discussed and reported the descriptive statistics, why don't you analyze the associations—at the very least, the most basic one, the chi square association—in this case?
Describe the dependent variable and the metrics used to measure it. For instance, what were the metrics used to evaluate the prenatal, institutional delivery, and postnatal care for each? The authors limit postnatal care, in particular, to 48 hours after delivery; however, they do not specify the number of visits that should take place during this time. The authors should also provide an explanation for their decision to use 48 hours rather than the 42-day postpartum period and at least 4 PNC visits.

**Answer:** We used the 48 hours postpartum period since it is the critical period window which can emphasize the most urgent phase for maternal and newborn interventions. The data we have are almost the same for both 48 hours and 42 days (low utilization of PNC services beyond 48 hours). We also compared our study with other studies that used 48 hours PNC visits.
**# Discussion section**
“A prior study on 29 Sub-Saharan African countries found an average proportion of women who had skilled assistance during delivery was 75.3%, ranging from 38.4% in Chad to 93.7% in Rwanda However, our study shows that only 13.4% of women in Somalia gave birth with the 10 help of skilled health provider.” The author attempts to compare their findings with those of previous studies. It would be preferable to compare with other comparable descriptive research because these studies are distinct from one another and cannot make sense when descriptive frequency-based results are compared to proportion-based findings.

**Answer:** We are comparing the descriptive figures from those countries like Chad (lowest) and Rwanda (highest) and we did not use the aggregate or average proportion figures. We have revisited this sentence too.
“The low utilization of SBA in Somalia can be improved by adopting prevailing successful interventions of countries with SBA successes such as Rwanda, whilst taking into account of contextual variations” needs reference citation

**Answer:** We have added the proper citations.
The authors stated that” The prevalence of maternal continuum of care in Somalia which is stated by our study (0.6%)” can the author put the confidence interval for this prevalence? I think it is a descriptive result and frequency not a “prevalence” furthermore, the authors stated that “The prevalence of maternal continuum of care in Somalia which is stated by our study (0.6%), is far much lower than the prevalence of continuum of care in Sub-Saharan Africa (25.0%), which varied from 17.9.0% in East Africa to 7,418 (51.5% in Southern Africa (27). It is also much lower than the 35.81% pooled prevalence of completion of the maternity continuum of care among 33 sub-Saharan African countries” It is not clear how to compare this one descriptive result with the pooled prevalence; instead, it would be appropriate to compare and discuss the studies with other comparable papers and setting outcomes.

**Answer:** We have revised this prevalence and proportions related figures and used similar papers.
I appreciate the authors' initiative and motivation, especially with regard to the crucial public health issue of the maternal health continuum of care in one of the areas with the highest rates of maternal
