# Supplementary material for: Level of completion of maternity continuum of care among ever-married women: An analysis of Somalia’s health and demographic survey 2020
Source: PLOS Glob Public Health. 2025 Jan 10;5(1):e0004102. doi: 10.1371/journal.pgph.0004102 (PMC11723541; doi:10.1371/journal.pgph.0004102)
Supplement: Attachment — Submitted filename: Response to Reviewers 2nd-Rebuttal.docx [file pgph.0004102.s005.docx]

PGPH-D-24-00010R1
Level of Completion of Maternity Continuum of Care among ever-married women: An analysis of Somalia Health and Demographic Survey 2020
PLOS Global Public Health

Dear Dr. Adam Abdulkadir Mohamed,

Thank you for submitting your manuscript to PLOS Global Public Health. After careful consideration, we feel that it has merit but does not fully meet PLOS Global Public Health’s publication criteria as it currently stands. Therefore, we invite you to submit a revised version of the manuscript that addresses the points raised during the review process.

Please submit your revised manuscript by Dec 13 2024 11:59PM. If you will need more time than this to complete your revisions, please reply to this message or contact the journal office at globalpubhealth@plos.org. When you're ready to submit your revision, log on to https://www.editorialmanager.com/pgph/ and select the 'Submissions Needing Revision' folder to locate your manuscript file.

Please include the following items when submitting your revised manuscript:

- A rebuttal letter that responds to each point raised by the editor and reviewer(s). You should upload this letter as a separate file labeled 'Response to Reviewers'.
- A marked-up copy of your manuscript that highlights changes made to the original version. You should upload this as a separate file labeled 'Revised Manuscript with Track Changes'.
- An unmarked version of your revised paper without tracked changes. You should upload this as a separate file labeled 'Manuscript'.

Guidelines for resubmitting your figure files are available below the reviewer comments at the end of this letter.

We look forward to receiving your revised manuscript.

Kind regards,

Damen Haile Mariam, MD, MPH, PhD
Academic Editor
PLOS Global Public Health

Journal Requirements:

Additional Editor Comments (if provided):

Reviewer 1:
- The manuscript, as a descriptive study, addresses a critical public health issue in Somalia, and meets PLOS Global Health criteria for publication. However, given the richness of the available data, incorporating a more in-depth inferential analysis could provide valuable insights by identifying factors or predictors associated with the outcome (maternal continuum of care).

**Answer:** This study, as it stands, is descriptive, and we did not intend to add determinants or correlations. This study is part of four manuscripts submitted for PhD thesis, so we made one paper on descriptives and another on correlates or predictors associated with the outcome.

Reviewer 2:
- General typographical comments:
- **Abstract section: The sentence “This study used the first ever DHS data in Somalia and did not get any funding” needs attention and rewritten with correct spelling.**

**Answer:** This is considered and re-corrected.
- **It would have been better if the authors had inserted page numbers and line numbers
- Affiliations:**

**Answer:** This is considered, and page numbers are inserted.

**- Author 2 and author 3 should write their department first in the affiliation.**

**Answer:** Adam A. Mohamed**^1,2^**, Ayşe Akın**^1^,** Sare Mihciokur^1^, Sarp Üner**^3^,** Abdi Gele^4^

**^1^** Department of Public Health, Faculty of Health Sciences, Başkent University, Ankara, Turkey

**^2^** Department of Program Quality & Development, Save the Children International, Mogadishu, Somalia

**^3^** Department of Public Health, Faculty of Medicine, Lokman Hekim University, Ankara, Turkey

**^4^** Department of Research, Norwegian Institute of Public Health, Oslo, Norway

***Adam Abdulkadir Mohamed** Correspondence: Adamafrican@gmail.com

Methods:
**- In your selection procedure it shows that “15,761 households interviewed” and “2,873 were not eligible.” After that it stated and showed that “12,888 eligible ever-married women aged 15-49” however, after interview “9,470 were not eligible” why? the authors shall clearly show the selection process for the scientific community it is confusing.**

**Answer:** 9,470 women were not eligible due to either needing to have children less than 5 years of age, incomplete data in the maternal and child health questions, or not fitting the targets, and we removed them during the data analysis.

**- In your Dependent and independent variables: Why did the authors list or display the independent and dependent variables? Since you have already discussed and reported the descriptive statistics, why don't you analyze the associations—at the very least, the most basic one, the chi-square association—in this case?**

**Answer:** We have removed the dependent and independent variables in the methods. This study, as it stands, is descriptive, and we did not intend to add determinants or correlations.

**Describe the dependent variable and the metrics used to measure it. For instance, what were the metrics used to evaluate the prenatal, institutional delivery, and postnatal care for each? The
authors limit postnatal care, in particular, to 48 hours after delivery; however, they do not specify the number of visits that should take place during this time. The authors should also provide an explanation for their decision to use 48 hours rather than the 42-day postpartum period and at least 4 PNC visits.**

**Answer:** We used the 48-hour postpartum period since it is the critical period window that can emphasize the most urgent phase for maternal and newborn interventions. Our data are almost the same for 48 hours and 42 days (low utilization of PNC services beyond 48 hours). We also compared our study with other studies that used 48-hour PNC visits.
- Discussion section
**- “A prior study on 29 Sub-Saharan African countries found an average proportion of women who had skilled assistance during delivery was 75.3%, ranging from 38.4% in Chad to 93.7% in Rwanda. However, our study shows that only 13.4% of women in Somalia gave birth with the 10 help of skilled health provider.” The author attempts to compare their findings with those of previous
studies. It would be preferable to compare with other comparable descriptive research because these studies are distinct from one another and cannot make sense when descriptive frequency-based results are compared to proportion-based findings.**

**Answer:** We are comparing the descriptive figures from countries like Chad (lowest) and Rwanda (highest), and we did not use the aggregate or average proportion figures. We have revisited this sentence in the study.

**- “The low utilization of SBA in Somalia can be improved by adopting prevailing successful interventions of countries with SBA successes such as Rwanda, whilst taking into account of contextual
variations” needs reference citation**

**Answer:** We have added the proper citations.

- The authors stated that” The prevalence of maternal continuum of care in Somalia which is stated by our study (0.6%)” can the author put the confidence interval for this prevalence? I think it is a
descriptive result and frequency not a “prevalence” furthermore, the authors stated that “The prevalence of maternal continuum of care in Somalia which is stated by our study (0.6%), is far much
lower than the prevalence of continuum of care in Sub-Saharan Africa (25.0%), which varied from 17.9.0% in East Africa to 7,418 (51.5% in Southern Africa (27). It is also much lower than the
35.81% pooled prevalence of completion of the maternity continuum of care among 33 sub-Saharan African countries” It is not clear how to compare this one descriptive result with the pooled
prevalence: instead, it would be appropriate to compare and discuss the studies with other comparable papers and setting outcomes.

**Answer:** We have revised this prevalence, and proportions related figures and used similar papers. Kindly see the paper in the discussion section.

Reviewer 3:

- General comment:
- The description presented in this study is similar with the reports of SDHS. It does not add new knowledge to the available evidence. However, the authors' interest with regard to the crucial public
health issue of continuum of care for improving maternal health is appreciated.

- Abstract:
**- Introduction: Somalia has one of the worst maternal conditions in the world with maternal mortality ratio of 692 per 100,000 live births. If maternal mortality is described, what is the importance of including a phrase “maternal conditions”? - Rephrase the sentence.**

**Answer:** We have revised and removed the maternal conditions.

- **Methods: There are inconsistences in the outcome stated in the objective and method. The outcome variable stated in the method section is only completion of maternal continuum of care.
However, the aim of the study is to investigate the coverage and the level of completion of continuum of maternity care. This needs aligning.**

**Answer:** We have revised the aim of the study to align with the methods section which is only completion of maternal continuum of care.

**- Results and Conclusion: The findings presented in results section had repetition and recommendation given in the conclusion section seems generic than based on the findings. Consider revision.**

**Answer:** We have expanded the recommendation section and added examples from similar contexts. “The government and partners should design and implement strategies to improve maternal healthcare utilization specific to rural and nomads, less educated, not working, low income, and less power to decision-making. The government of Somalia should invest and increase the number of certified skilled birth attendants i.e., nurses, midwives, and obstetricians to provide safe and competent care during childbirth. To attract and retain skilled birth attendants in remote and rural areas, the government and its partners should implement incentive programs designed to the professional and certified midwives and obstetricians and should train and deploy community health extension workers in rural areas to provide basic maternal health educations, identify pregnant women, and support and encourage them to seek health care”.

**- Introduction:
- The introduction section was more focused on status of maternal health in Somalia than utilization of continuum of care.
- Concept of Continuum of care was not well presented. The authors need to provide definition they used for “Continuum of care”, from available definitions in the literature.
- A paragraph written on continuum of care has redundancy of phrases.
- Limited evidence was presented on status utilization of continuum of care (globally, regionally or nationally). The gap that was addressed by this study was not clearly presented. This section
needs a major revision including addition of literature review and revision of the available evidence.**

**Answer:** We have completely rewritten the introduction section and added sections on the utilization of the continuum of care. We added the definition of CoC used in this study. We have rephrased the gap in the study.

**- Methods:
- It is very brief and not well described.
- Sampling procedure of the study participants was not well described.**

**Answer:** We have added explanations on sampling of the study participants.

- **The procedure followed to access the data was not described and may have ethical concerns.
- Why were 9470 women not eligible in Figure 1? - Include the reason and make it self-explanatory.**

**Answer:** 9,470 women were not eligible due to either needing to have children less than 5 years of age, incomplete data in the maternal and child health questions, or not fitting the targets, and we removed them during the data analysis.

- Methods used for management of missed data was not explained.

**Answer:** We have completely rewritten the methods section again and addressed all of the suggestions. We have added a deep explanation of the missing data in the data analysis section.

- What is the implication of this sentence «Marriage is associated with childbearing and women can have a child only if she is married as accepted in the community”? How was marriage defined in
the context of this sentence? What is the relation of marriage with child-birth? The authors have to describe it in detail.

**Answer:** We have added an explanation of restricting the analysis to ever-married women in Somalia. Marriage is associated with childbearing and women can have a child only if she is married as accepted in the community. That is why we restricted our study on only the ever-married women. In the Somalia context, all women who have children had marriage previously, which means women cannot have a child without legal marriage.

- Results:
- The terms prevalence, proportion, inequality, and bracket were not appropriately used.
- Use of ANC versus antenatal care, SBA versus skilled birth attendant, and PNC versus postnatal care needs to be standardized/uniform.
- Table headings are placed wrongly.

**Answer:** This is well noted and we have addressed and used a unified wording and abbreviations.

- Discussion:
- Did not follow the standard ways of presenting major findings. The findings were not discussed with relevant evidence from the literature
- Many limitations in the methods section were not mentioned under the limitations section.
- Some information is redundant and can be taken out or shortened.

**Answer:** We managed to re-write the discussion section too. We have enriched examples of policies and strategies in the manuscript.

**- Conclusion:
- Not comprehensively presented and it needs to be summarized**

**Answer:** We have expanded the conclusion section.  **- Recommendations are generic which are not based on the finding of the study.**

**Answer:** We have expanded the recommendation section and added examples from similar contexts. “The government and partners should design and implement strategies to improve maternal healthcare utilization specific to rural and nomads, less educated, not working, low income, and less power to decision-making. The government of Somalia should invest and increase the number of certified skilled birth attendants i.e., nurses, midwives, and obstetricians to provide safe and competent care during childbirth. To attract and retain skilled birth attendants in remote and rural areas, the government and its partners should implement incentive programs designed to the professional and certified midwives and obstetricians and should train and deploy community health extension workers in rural areas to provide basic maternal health educations, identify pregnant women, and support and encourage them to seek health care”.

**- References:
- Check referencing style - for instance, Ref.no. 5.**

**Answer:** We have addressed and corrected.

**Minor Issues:**

1. The citations in the main manuscript should be revised to align with PLOS Global Health referencing style.

**Answer:** We have aligned the citations with PLOS Global Health referencing style. We used the Vancouver referencing style.

2. There are minor grammatical errors throughout the manuscript. A thorough proofreading is recommended to enhance readability.

**Answer:** We have made a line-by-line proofreading.

3. The references are generally well-cited, but incorporating more recent studies on the maternity continuum of care and maternal health in conflict settings would strengthen the manuscript's academic rigor.

**Answer:** Added more recent studies in the manuscript.

4. Some references are inaccurately attributed. For example, the manuscript incorrectly states, "This rate is much higher than the completion of ANC4 in Uganda (59.9%) ...".

**Answer:** We have corrected the inaccurate figures and sentences.

5. The citations in the manuscript should be revised to comply with the PLOS Global Health referencing style.

**Answer:** We have revised the citations.
